# Coordination Chemistry at the Hard–Soft Interface: Phosphine Oxide-Based Rare Earth/Transition Metal Complexes

**DOI:** 10.1021/acsomega.5c13312

**Published:** 2026-02-22

**Authors:** Rwitabrita Panda, Franziska Flecken, Christina Papke, Christopher E. Anson, Toni Grell, Schirin Hanf

**Affiliations:** † Institute for Inorganic Chemistry, 150232 Karlsruhe Institute of Technology, Engesserstr. 15, Karlsruhe 76131, Germany; ‡ Dipartimento di Chimica, 9304 Università Degli Studi diMilano, Via Camillo Golgi 19, Milan 20131, Italy

## Abstract

A series of monometallic Al­(III), Sm­(III), Dy­(III), Er­(III), and Yb­(III) complexes, featuring tetraphenyldiphosphine monoxide (PPO) as a ligand, were synthesized and characterized. These complexes served as precursors for the construction of heterobimetallic rare earth (RE)/transition metal (TM) assemblies. Attempts to introduce soft TMs, such as Cu­(I) and Au­(I), into the preformed RE–PPO synthons predominantly afforded equilibrium-driven TM-based POP species, underscoring the challenges of incorporating hard and soft metal centers directly. This observation led to an alternative route employing a presynthesized Mo–PPO synthon, which led to the successful formation of RE/Mo heterobimetallic complexes upon addition of the RE ions. The molecular structures of both mono- and heterobimetallic species were strongly influenced by the solvent environment. Notably, reactions in dichloromethane, a noncoordinating solvent, yielded RE_2_ complexes featuring chloride bridges, a motif absent when coordinating solvents, such as THF or MeCN were employed. This solvent-dependent structural divergence offers a straightforward strategy for modulating the metal nuclearity within the complexes.

## Graphical abstract

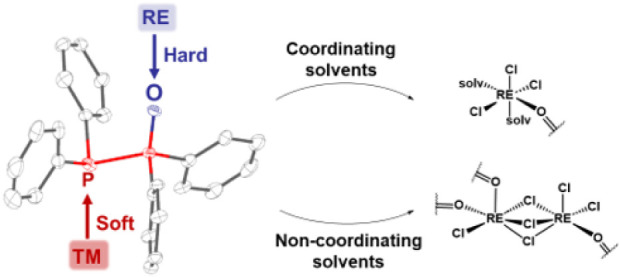

## Introduction

The study of homo-, hetero-, and multimetallic transition metal (TM) complexes has been a cornerstone in catalysis and small molecule activation-related research.^1^ A key advantage of these systems is their potential cooperativity, meaning two or more metal centers act in concert, either simultaneously or consecutively, to enhance reactivity.^1^ While such cooperativity is well-documented for early–late TM complexes, such as Zr–Ru, Fe–Cu, Zr–Co, and many more,^2–4^ the incorporation of rare earth (RE) metals into heterobimetallic architectures has received considerably less attention. This is due to the fact, that synthetic attempts to synthesize RE/TM complexes, often only result in the isolation of homometallic TM complexes.^5^

Pioneering studies by Beletskaya, Kempe, Nippe and coworkers established the existence of direct, unsupported RE–TM bonds in Y/Yb/Lu–Ru,^6–8^ RE–Re (RE = Y, La, Sm, Yb, Lu),^6,7,9,10^ and Dy–Fe/Ru^11^ complexes. Subsequent advances by Diaconescu, Roesky, Lu, Cui, and Hlina have significantly expanded the field by developing tailored ligand frameworks that can simultaneously bind soft transition metals and hard rare earth centers (Scheme 1). Notable examples include ferrocene-based scaffolds,^12^ as well as phosphinoamido frameworks that stabilize Y/Lu–Pt/Pd,^13^ RE–Ni^14–16^ and Sc–Pd^17^ interactions. Hlina and coworkers only recently extended the field toward phosphinophenolate ligands for the synthesis of RE/Ag and RE/Cu complexes with weak RE–TM interactions.^18,19^ P,O-based ligands^20^ were also reported by Hepiegne for the synthesis of U/TM (TM = W, Mo, Ru) complexes, however without any direct metal–metal interactions.^21^ Arnold *et al.* extended the field with uranium–group 10 metal complexes with varying U–TM bonding strengths.^22^ Although such RE-based heterobimetallic complexes show promising applications in homogeneous^12^ ,^14^ ,^15^ ,^23^ ,^24^ and heterogeneous catalysis,^25,26^ photophysics,^27,28^ and as single-molecule magnets (SMMs),^29–31^ their controlled synthesis remains a significant challenge.

**Scheme 1:**
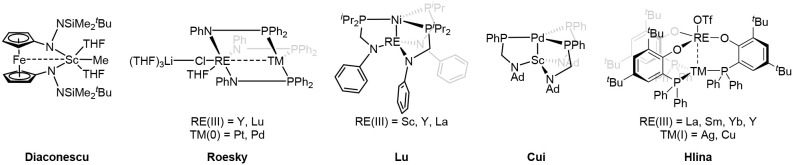
Selected examples of ligand-supported heterobimetallic complexes.

A promising strategy for assembling RE and TM ions within a single complex is guided by the Hard Soft Acid Base (HSAB) principle, which enables a rational ligand design for bridging hard and soft metal centers.^32^ This concept has been realized through three main synthetic approaches. The first involves preorganized, compartmentalized ligands, such as Schiff bases, that provide distinct coordination environments for each metal center.^33,34^ The second approach employs assisted self-assembly, in which selective metal–ligand interactions direct the spontaneous organization of RE and TM ions.^35–39^ The third strategy adopts a site-targeted substitution methodology, wherein one metal center in a self-assembled complex is selectively exchanged to probe the cooperative influence between the two metals.^40,41^ Collectively, these approaches highlight that the successful construction of RE/TM complexes is critically dependent on the choice of ligand architecture. Within this context, multidentate mixed-donor phosphine ligands and their transition-metal complexes are of particular importance, especially in homogeneouslycatalyzed reactions, such as hydrogenation, hydroformylation, and coupling reactions.^42–44^ Their effectiveness often arises from their hemilabile behavior, where reversible coordination and decoordination of donor groups transiently generate vacant sites for substrate binding.

Building on these design principles, our group has focused on exploring tetraphenyldiphosphine monoxide (Ph_2_PP­(=O)­Ph_2_, PPO) as a versatile multidentate mixed-donor ligand capable of coordinating to both hard and soft metal centers. We have previously reported its rich coordination chemistry toward a variety of transition metals.^45–47^ A fascinating aspect of the PPO ligand is its tautomeric equilibrium, a so-called phosphorotropic tautomerism, with tetraphenyldiphosphoxane (Ph_2_P–O–PPh_2_, POP, Figure 1). While density functional theory (DFT) indicates that the PPO tautomer is thermodynamically favored, except when the phenyl substituents are replaced by strong electron-withdrawing groups (e.g., CF_3_ or 2,4-CF_3_–C_6_H_3_),^48–50^ the equilibrium can be shifted through metal coordination. Interactions with soft transition metals, such as Cu­(I) and Au­(I), promoted the formation of POP-based complexes, including [Cu_2_(MeCN)_*x*_(μ_2_-POP)_2_]­(PF_6_)_2_ (*x* = 2,3,4), [Au_2_Cl_2_(μ_2_-POP)] and [Au_2_(μ_2_-POP)_2_]­(OTf)_2_. In contrast, coordination with hard metals, such as Fe­(III) and Y­(III), led to the formation of PPO-type complexes, [FeCl_2_(PPO)_2_] and [YCl_3_(THF)_2_(PPO)], where the hard oxygen donor preferentially binds the metal center.^45^

**Figure 1:**
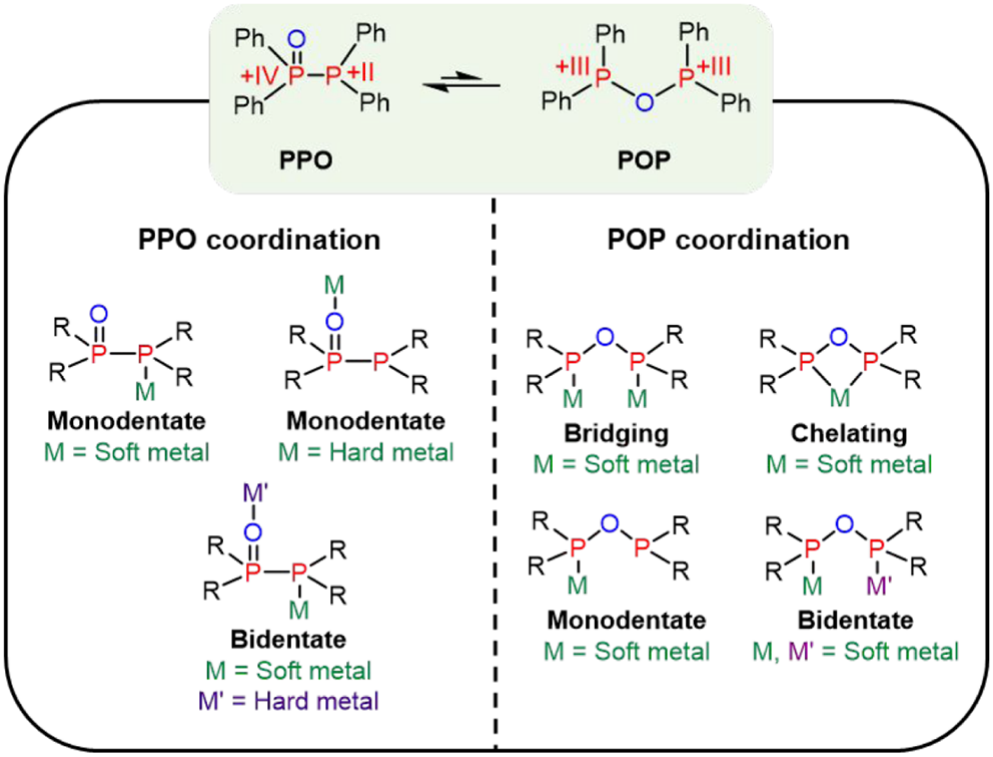
Phosphorotropic tautomerism of the PPO/POP ligand, illustrating its various potential coordination modes.

Among soft transition metals, molybdenum represents a particularly interesting candidate for coordination studies with the PPO/POP ligand scaffold, owing to its ability to adopt multiple coordination modes that can be finely tuned by the reaction conditions, such as solvent and temperature. Mo(0) can engage with the PPO/POP system in monodentate, chelating, or bridging fashions (Figure 2). In the monodentate mode, Mo(0) coordinates either to the P­(II) donor side of PPO or the P­(III) donor side of the POP tautomer, forming [Mo­(CO)_5_(κ*P*-PPO)][Bibr ref51] or [Mo­(CO)_5_(κ*P*-POP)],^52^ respectively. When Mo­(CO)_6_ is heated with PPO, a chelating coordination mode can be adopted, affording *cis*-[Mo­(CO)_4_(κ^2^ *P*,*P*′-POP)]. The same chelating motif can also be accessed *via* reactions of *cis*-[Mo­(PPh_2_O)_2_H]^−^ with chlorophosphines or acyl chlorides.^52^ In addition, Mo(0) can coordinate in a bridging coordination mode to POP, forming [Mo­(CO)_5_{μ-POP}­Mo­(CO)_5_].^53^

**Figure 2:**
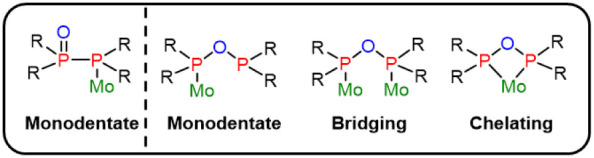
Coordination diversity of molybdenum within the PPO/POP ligand framework.

On the basis of these previous findings, we report the use of the multidentate mixed-donor PPO/POP ligand scaffold for the synthesis of heterobimetallic rare earth/molybdenum complexes. In this strategy, the soft phosphorus donor sites are tailored to coordinate the soft Mo(0) centers, whereas the hard oxygen donors preferentially bind the hard RE­(III) ions. By exploiting the ambidentate and adaptive coordination behavior of the PPO/POP ligand set, this approach enables the construction of new molecular architectures, that integrate the complementary electronic characteristics of RE and Mo centers.

## Experimental Section

### Materials, Methods, and Instruments

All experiments were carried out on a Schlenk-line under Ar atmosphere or in an Ar-filled glovebox (MBraun). Toluene, tetrahydrofuran (THF), *n*-pentane and *n*-heptane were dried using an MBraun solvent purification system (SPS-800) and degassed. THF was additionally distilled under Ar from potassium benzophenone before storage over 4 Å molecular sieves. Acetonitrile (MeCN), dichloromethane (DCM) and triethylamine were distilled over calcium hydride. CDCl_3_ and CD_2_Cl_2_ were dried over P_2_O_5_, while C_6_D_6_, toluene-d_8_ and CD_3_CN were dried over CaH_2_. Deuterated solvents were degassed by freeze–pump–thaw cycles prior to use. All solvents were stored over activated molecular sieves (MeCN over 3 Å, all other solvents over 4 Å). Chlorodiphenylphosphine was purified by distillation. All other chemicals were used without further purification. Ph_2_PP­(=O)­Ph_2_ (PPO)^45^ and [Mo­(CO)_5_(PPO)] (MoPPO)^51^ were prepared following literature procedures.

Nuclear magnetic resonance (NMR) spectra were recorded on a Bruker AVANCE III or Avance Neo 400 MHz spectrometer at 298 K. Chemical shifts are given in ppm and are referenced on residual solvent signals of deuterated solvents. Unambiguous assignments were determined on the basis of chemical shifts, coupling patterns and 2D NMR experiments (^1^H–^13^C HSQC, ^1^H–^13^C HMBC). The multiplicity of the NMR signals is denoted as s = singlet, d = doublet, dd = doublet of doublets, t = triplet, q = quartet, sep = septet, m= multiplet, and br = broad. The ^31^P­{^1^H} NMR peak assignments for the PPO ligand are provided in the Supporting Information (Figure S1).

Infrared (IR) spectra were recorded, in the region of 4000–400 cm^–1^, on a Bruker Tensor 37 FTIR spectrometer equipped with a room temperature DLaTGS detector, a diamond ATR (attenuated total reflection) unit and a nitrogen-flushed measurement chamber. IR signals were classified according to their intensities (vs = very strong, m = medium, w = weak, vw = very weak).

Elemental Analyses were carried out with an Elementar vario MICRO Cube device.

Single crystal X-ray diffraction (SC-XRD) data sets were measures on a STOE STADIVARI diffractometer equipped with a MoGenix 3D HF X-ray source (Mo–Kα radiation, λ = 0.71073 Å). Single crystals suitable for X-ray diffraction were transferred directly from the mother liquor to perfluoropolyalkylether oil, mounted on MiTeGen MicroMounts and rapidly transferred to the diffractometer, where the crystals were cooled to 100 K using an Oxford Cryostream. Data acquisition, unit cell determination, and integration were carried out using the STOE X-Area 2.4 software. Absorption corrections were applied using Gaussian integration as implemented in STOE X-Red32 2.3.1,^54^ followed by scaling of reflection intensities using STOE LANA 2.8.4 ^55^ integrated within the X-Area program. The structures were solved by dual-space methods using SHELXT^56^ and refined by full-matrix least-squares methods against *F* ^2^ using SHELXL-2019/3 ^57^ within the Olex2 1.5 ^58^ platform. All non-hydrogen atoms were refined anisotropically, and hydrogen atoms were placed in calculated positions with a riding model. In **2** and **3** one or both of the PPO ligands, respectively, showed minor (7%, 8%, 15%) disorder of their P_2_Ph_4_ units. In each case, only the P atoms of the minor components could be located, and no attempt was made to split the corresponding phenyl groups. Similarly in **10,** the [(CO)_5_Mo­(PPO)] unit involving Mo(2) showed minor (9%) disorder of the outer PPh_2_Mo­(CO)_5_ unit, and again only the P- and Mo-atoms of the minor component could be located. In **4** and **5** the clathrated THF molecules are disordered over an inversion center and could not be refined satisfactorily. In **10** one CH_2_Cl_2_ molecule in the lattice was ordered and could be refined anisotropically, but two further CH_2_Cl_2_ molecules and a *n*-pentane molecule were heavily disordered. The contributions of these disordered solvent molecules to the structure factors were calculated using SQUEEZE,^59^ and the formulas given in Table S2 include these molecules. Graphic representations of the molecular structures have been prepared with Diamond^60^ and in all figures hydrogen atoms have been omitted for clarity, and ellipsoids are depicted at the 30% probability level. Full crystallographic data and details of the structural determinations for the structures in this paper have been deposited with the Cambridge Crystallographic Data Centre as supplementary publication nos. CCDC 2488821–2488830. Copies of the data can be obtained, free of charge, from https://www.ccdc.cam.ac.uk/structures/.

UV–visible spectra were recorded using an Ocean FX spectrometer (Ocean Optics) with solutions of concentrations 1 × 10^–3^ M prepared in THF solution. The resulting data were processed in Origin 2023.

### Syntheses

#### Synthesis of [AlCl_3_(PPO)] (1)

Anhydrous AlCl_3_ (34.5 mg, 0.26 mmol) and PPO (100.0 mg, 0.26 mmol) were dissolved in THF (5 mL) and stirred for 2 h. The solvent was then reduced to approximate 3 mL and after layering with *n*-heptane, colorless crystals of **1** suitable for single crystal X-ray analysis were obtained. Crystalline yield: 58.0 mg, 0.11 mmol, 43%. ^1^H NMR (298 K, CDCl_3_, 400 MHz) δ [ppm] 7.76–7.67 (4H, m, P­(IV)–Ph–H-*ortho*), 7.65–7.58 (2H, m, P­(IV)–Ph–H-*para*), 7.58–7.40 (10H, m, P­(IV)–Ph–H-*meta*, P­(II)–Ph–H-*ortho*, *para*), 7.40–7.32 (4H, m, P­(II)–Ph–H-*meta*). ^31^P­{^1^H} NMR (298 K, CDCl_3_, 162 MHz) δ [ppm] = 57.4 (d, P­(IV), ^1^ *J*_PP_ = 310.2 Hz), −17.84 (d, P­(II), ^1^ *J*_PP_ = 310.3 Hz). ^13^C­{^1^H} NMR (298 K, CDCl_3_, 101 MHz), δ [ppm] = 135.6–135.2 (m, P­(II)–Ph–C-*ortho*), 134.1–133.9 (br, s, P­(IV)–Ph–C-*ipso*), 132.0–131.7 (m, P­(IV)–Ph–C-*ortho*, *para*), 131.5–131.3 (br, s, P­(II)–Ph–C-*para*), 129.6–129.3 (m, P­(II)–Ph–C-*ipso*, *meta*, P­(IV)–Ph–C-*meta*). Elemental analysis, calcd for [AlCl_3_(PPO)] C 55.47, H 3.88; found C 54.08, H 3.87. ATR-IR [cm^–1^] 3054 (vw), 1587 (vw), 1481 (vw), 1436 (w), 1392 (vw), 1336 (vw), 1311 (vw), 1128 (m), 1114 (s), 1070 (s), 1024 (w), 997 (w), 937 (vw), 918 (vw), 848 (vw), 738 (m), 688 (s), 619 (vw), 601 (vw), 561 (w), 503 (vs), 466 (m), 445 (w), 432 (w).

#### Synthesis of [SmCl_3_(PPO)_2_(THF)] (2)

SmCl_3_ (33.2 mg, 0.13 mmol) and PPO (100.0 mg, 0.26 mmol) were dissolved in THF (5 mL) and stirred for 2 h. The solution was filtered and after layering with *n*-heptane colorless crystals of **2** suitable for single-crystal X-ray diffraction analysis could be grown. Crystalline yield: 32.0 mg, 0.03 mmol, 23%. ^1^H NMR (298 K, THF-*d*_8_, 400 MHz), δ [ppm] = 8.28–8.10 (8H, br, s, P­(IV)–Ph–H-*ortho*), 7.66–7.52 (8H, m, P­(II)–Ph–H-*ortho*), 7.50–7.35 (12H, m, P­(IV)–Ph–H-*para*, *meta*), 7.29–7.17 (12H, m, P­(II)–Ph–H-*meta*, *para*). ^31^P­{^1^H} NMR (298 K, THF-*d*_8_, 162 MHz), δ [ppm] = 43.5–36.3 (br, m, P­(IV)), −21.4 (d, P­(II),^1^ *J*_PP_ = 249.7 Hz). ^13^C­{^1^H} NMR (298 K, THF-*d*_8_, 101 MHz), δ [ppm] = 136.1–135.4 (m, P­(II)–Ph–C-*ortho*, P­(IV)–Ph–C-*ipso*), 132.2–131.7 (m, P­(IV)–Ph–C-*ortho, para*), 129.7–129.5 (br, s,P­(II)–Ph–C-*para*), 128.5–128.1 (m, P­(II)–Ph–C-*ipso*, *meta*, P­(IV)–Ph–C-*meta*). Elemental analysis, calcd for [SmCl_3_(PPO)_2_] C 56.0, H 3.92; found C 56.43, H 4.20. ATR-IR [cm^–1^] 3853 (vw), 3054 (w), 2985 (vw), 1477 (vw), 1434 (s), 1126 (vs), 1070 (vs), 1026 (w), 1010 (w), 997 (w), 854 (w), 740 (s), 721 (w), 690 (vs), 561 (m), 513 (w), 501 (s), 486 (w), 460 (w), 437 (vw).

#### Synthesis of [DyCl_3_(CH_3_CN)­(PPO)_2_] (3)

DyCl_3_ (34.8 mg, 0.13 mmol) and PPO (100.0 mg, 0.26 mmol) were dissolved in MeCN (5 mL) and stirred for 2 h. The solution was then filtered followed by solvent reduction to approximate 3 mL and allowed to stand at room temperature. Colorless crystals of **3** suitable for single crystal X-ray analysis were obtained. Crystalline yield: 43.0 mg, 0.04 mmol, 31%. The poor quality of the NMR spectra corresponds to the paramagnetic nature of the Dy­(III) center. Elemental analysis, calcd for [DyCl_3_(CH_3_CN)­(PPO)_2_] C 55.47, H 4.00; found C 54.82, H 4.38. ATR-IR [cm^–1^] 3054 (vw), 1650 (vw), 1616 (vw), 1587 (vw), 1558 (vw), 1540 (vw), 1479 (vw), 1434 (m), 1400 (vw), 1334 (vw), 1315 (vw), 1186 (vw), 1130 (vs), 1083 (s), 1070 (s), 1026 (w), 997 (w), 925 (vw), 740 (m), 721 (w), 690 (vs), 617 (vw), 559 (s), 505 (vs), 460 (w), 435 (w).

#### Synthesis of [ErCl_3_(PPO)­(THF)_2_] (4)

ErCl_3_ (70.8 mg, 0.26 mmol) and PPO (100.0 mg, 0.26 mmol) were dissolved in THF (5 mL) and stirred for 2 h. The solution was then filtered followed by solvent reduction to approximate 3 mL. After layering with *n*-heptane, colorless crystals of **4** suitable for single crystal X-ray analysis were obtained. Crystalline yield: 22.0 mg, 0.03 mmol, 11%. The poor quality of the NMR spectra corresponds to the paramagnetic nature of the Er­(III) center. Elemental analysis, calcd for [ErCl_3_(PPO)­(THF)_2_] C 47.79, H 4.51; found C 47.28, H 4.50. ATR-IR [cm^–1^] 1436 (vw), 1305 (w), 1232 (s), 1201 (m), 1184 (m), 1130 (vs), 1085 (m), 1070 (w), 1056 (w), 1012 (w), 981 (m), 918 (vw), 862 (w), 810 (vw), 742 (w), 721 (w), 694 (m), 640 (vw), 557 (m), 514 (m), 499 (s), 468 (vw), 437 (vw).

#### Synthesis of [YbCl_3_(PPO)­(THF)_2_] (5)

YbCl_3_ (72.3 mg, 0.26 mmol) and PPO (100.0 mg, 0.26 mmol) were dissolved in THF (5 mL) and stirred for 2 h. The solution was then concentrated to 3 mL and then filtered. After layering the filtrate with *n*-heptane colorless crystals of **5** suitable for single crystal X-ray analysis were obtained. Crystalline yield: 44.0 mg, 0.05 mmol, 21%. The poor quality of the NMR spectra corresponds to the paramagnetic nature of the Yb­(III) center. Elemental analysis, calcd for [YbCl_3_(PPO)­(THF)_2_] C 48.12, H 4.77; found C 48.12, H 4.50. ATR-IR [cm^–1^] 3054 (vw), 2973 (w), 2937 (vw), 2900 (vw), 2871 (vw), 1481 (vw), 1436 (m), 1311 (w), 1240 (m), 1184 (w), 1137 (vs), 1107 (m), 1087 (s), 1072 (m), 1039 (w), 1014 (m), 995 (w), 919 (vw), 860 (m), 756 (vw), 742 (s), 719 (w), 696 (s), 559 (w), 516 (w), 499 (m), 470 (vw), 437 (vw).

#### Synthesis of [(PPO)­Cl_2_Y­{μ-Cl_3_}­YCl­(PPO)_2_] (6)

YCl_3_ (25.0 mg, 0.13 mmol) and PPO (50.0 mg, 0.13 mmol) were dissolved in DCM (3 mL) and stirred for 2 h. The white solution was filtered and after layering with *n*-heptane colorless crystals of **6** suitable for single-crystal X-ray diffraction analysis could be grown. Crystalline yield: 124.0 mg, 0.08 mmol, 62%. ^1^H NMR (298 K, CD_2_Cl_2_, 400 MHz), δ [ppm] = 8.01–7.91 (4H, m, P_1_(IV)–Ph–H-*ortho*), 7.78–7.70 (4H, m, P_2_(II)–Ph–H-*ortho*), 7.70–7.55 (16H, m, P_3,5_(IV), P_4,6_(II)–Ph–H-*ortho*), 7.55–7.50 (2H, m, P_1_(IV)–Ph–H-*para*), 7.48–7.40 (4H, m, P_1_(IV)–Ph–H-*meta*), 7.40–7.32 (10H, m, P_3,5_(IV)–Ph–H-*para*, P_2_(II)–Ph–H-*meta, para*), 7.32–7.20 (12H, m, P_3,5_(IV)–Ph–H-*meta*, P_4,6_(II)–Ph–H-*para*), 7.14–7.05 (8H, m, P_4,6_(II)–Ph–H-*meta*). ^31^P­{^1^H} NMR (298 K, CD_2_Cl_2_, 162 MHz), δ [ppm] = 52.0 (dd, P_3,5_(IV),^1^ *J*_PP_ = 284.8 Hz, ^2^ *J*_PY_ = 10.6 Hz), 51.0 (dd, P_1_(IV),^1^ *J*_PP_ = 284.8 Hz, ^2^ *J*_PY_ = 10.6 Hz), −16.1 (d, P_4,6_(II),^1^ *J*_PP_ = 284.8 Hz), −18.6 (d, P_2_(II),^1^ *J*_PP_ = 284.8 Hz). ^13^C­{^1^H} NMR (298 K, CD_2_Cl_2_, 101 MHz), δ [ppm] = 136.2–135.5 (m, P_2_(II)–Ph–C-*ortho*, P_4,6_(II)–Ph–C-*ortho*, P_1_(IV)–Ph–C-*ipso*, P_3,5_(IV)–Ph–C-*ipso*), 132.9–132.7 (br, s, P_3,5_(IV)–Ph–C-*para*, P_2_(II)–Ph–C-*para*), 132.1–131.7 (m, P_1_(IV)–Ph–C-*ortho*, P_3,5_(IV)–Ph–C-*ortho*, P_1_(IV)–Ph–C-*para*), 130.7–130.2 (m, P_2_(II)–Ph–C-*ipso*, P_4,6_(II)–Ph–C-*ipso*), 129.4–129.1 (m, P_3,5_(IV)–Ph–C-*meta*, P_4,6_(IV)–Ph–C-*para*), 128.8–128.4 (m, P_1_(IV)–Ph–C-*meta* P_4,6_(II)–Ph–C-*meta,* P_2_(II)–Ph–C-*meta*). Elemental analysis, calcd for [(PPO)­Cl_2_Y­{μ-Cl_3_}­YCl­(PPO)_2_] C 55.81, H 3.90; found C 55.55, H 3.90. ATR-IR [cm^–1^] 3853 (vw), 3745 (vw), 3056 (w), 1434 (vs), 1118 (s), 1078 (s), 1060 (vs), 1026 (w), 999 (w), 742 (m), 721 (w), 688 (vs), 563 (m), 513 (m), 501 (s), 462 (w).

#### Synthesis of [YCl_3_(PPO)­(THF)_2_Mo­(CO)_5_] (7)

YCl_3_ (31.4 mg, 0.16 mmol) and MoPPO (100.0 mg, 0.16 mmol) were dissolved in THF (5 mL) and stirred for 2 h. The yellow solution was then filtered and after layering with *n*-heptane small brick red crystals of **7** suitable for single X-ray diffraction were obtained. Crystalline yield: 64.0 mg, 0.07 mmol, 41%. ^1^H NMR (298 K, CDCl_3_, 400 MHz) δ [ppm] 7.79–7.67 (4H, m, P­(IV)–Ph–H-*ortho*), 7.65–7.56 (4H, m, P­(II)–Ph–H-*ortho*), 7.54–7.12 (12H, m, P­(IV)–Ph–H-*meta, para*, P­(II)–Ph–H-*meta*, *para*). ^31^P­{^1^H} NMR (298 K, CDCl_3_, 162 MHz) δ [ppm] = 51.9–48.1 (m, P­(IV)), 44.3 (d, P­(II), ^1^ *J*_PP_ = 146.8 Hz). ^13^C­{^1^H} NMR (298 K, CDCl_3_, 101 MHz), δ [ppm] = 135.0–134.6 (m, P­(II)–Ph–C-*ortho*), 134.3–134.1 (br, s, P­(IV)–Ph–C-*ipso*), 133.9–133.6 (m, P­(IV)–Ph–C-*ortho*, *para*), 131.7–131.5 (br, s, P­(II)–Ph–C-*para*), 129.4–128.6 (m, P­(II)–Ph–C-*ipso*, *meta*, P­(IV)–Ph–C-*meta*). Elemental analysis, calcd for [YCl_3_(PPO)­(THF)_2_Mo­(CO)_5_] C 46.20, H 3.77; found C 46.24, H 3.71. ATR-IR [cm^–1^] 3340 (s), 3238 (w), 3097 (vw), 3078 (vw), 3056 (vw), 3024 (vw), 2071 (w), 1955 (m), 1936 (vs), 1917 (vs), 1635 (w), 1620 (w), 1587 (vw), 1575 (vw), 1558 (vw), 1540 (vw), 1521 (vw), 1506 (vw), 1479 (vw), 1458 (vw), 1436 (vw), 1176 (vw), 1157 (vw), 1130 (vw), 1103 (vw), 1087 (vw), 1072 (vw), 1026 (vw), 1012 (vw), 997 (vw), 929 (vw), 860 (vw), 748 (vw), 694 (vw), 601 (vw), 582 (vw), 561 (vw), 524 (vw), 513 (vw), 497 (vw), 486 (vw), 460 (vw), 422 (vw).

#### Synthesis of [DyCl_3_(PPO)­(THF)_2_Mo­(CO)_5_] (8)

DyCl_3_ (43.2 mg, 0.16 mmol) and MoPPO (100.0 mg, 0.16 mmol) were dissolved in THF (5 mL) and stirred for 2 h. The yellowish colored solution was then filtered and after layering with *n*-heptane tiny pale-yellow floral like crystals of **8** suitable for single crystal X-ray analysis were obtained. Crystalline yield: 30.0 mg, 0.03 mmol, 18%. The poor quality of the NMR spectra corresponds to the paramagnetic nature of the Dy­(III) center. Elemental analysis, calcd for [DyCl_3_(PPO)­(THF)_2_Mo­(CO)_5_] C 42.92, H 3.50; found C 42.84, H 3.41. ATR-IR [cm^–1^] 3340 (m), 3232 (w), 3097 (vw), 3078 (vw), 3058 (vw), 2071 (w), 1992 (vw), 1934 (vs), 1917 (vs), 1683 (vw), 1633 (w), 1618 (w), 1587 (vw), 1575 (vw), 1558 (vw), 1540 (vw), 1521 (vw), 1506 (vw), 1479 (vw), 1458 (vw), 1436 (vw), 1311 (vw), 1176 (vw), 1157 (vw), 1128 (vw), 1105 (vw), 1087 (vw), 1074 (vw), 1026 (vw), 1012 (vw), 997 (vw), 860 (vw), 748 (vw), 721 (vw), 694 (vw), 601 (vw), 582 (vw), 561 (vw), 524 (vw), 513 (vw), 497 (vw), 486 (vw), 460 (vw), 422 (vw).

#### Synthesis of [LuCl_3_(PPO)­(THF)_2_Mo­(CO)_5_] (9)

LuCl_3_ (45.2 mg, 0.16 mmol) and MoPPO (100.0 mg, 0.16 mmol) were dissolved in THF (5 mL) and stirred for 2 h. The yellowish colored solution was then filtered and after layering with *n*-heptane tiny yellow flower-like crystals of **9** suitable for single crystal X-ray diffraction were obtained. Crystalline yield: 91.2 mg, 0.09 mmol, 54%. ^1^H NMR (298 K, CDCl_3_, 400 MHz) δ [ppm] 7.87–7.72 (4H, m, P­(IV)–Ph–H-*ortho*), 7.72–7.59 (4H, m, P­(II)–Ph–H-*ortho*), 7.59–7.14 (12H, m, P­(IV)–Ph–H-*meta, para*, P­(II)–Ph–H-*meta*, *para*). ^31^P­{^1^H} NMR (298 K, CDCl_3_, 162 MHz) δ [ppm] = 57.4 (d, P­(IV),^1^ *J*_PP_ = 310.2 Hz), −17.8 (d, P­(II), J = 310.3 Hz. ^13^C­{^1^H} NMR (298 K, CDCl_3_, 101 MHz), δ [ppm] = 135.1–134.8 (m, P­(II)–Ph–C-*ortho*), 134.4–134.3 (br, s, P­(IV)–Ph–C-*ipso*), 134.1–133.8 (m, P­(IV)–Ph–C-*ortho*, *para*), 131.6–131.4 (br, m, P­(II)–Ph–C-*para*), 129.3–128.2 (m, P­(II)–Ph–C-*ipso*, *meta*, P­(IV)–Ph–C-*meta*). Elemental analysis, calcd for [LuCl_3_(PPO)­(THF)_2_Mo­(CO)_5_] C 42.41, H 3.46; found C 42.41, H 3.45. ATR-IR [cm^–1^] 3066 (vw), 2077 (w), 2007 (vw), 1953 (vs), 1926 (vs), 1772 (vw), 1751 (vw), 1733 (vw), 1716 (vw), 1699 (vw), 1683 (vw), 1652 (vw), 1585 (vw), 1479 (vw), 1456 (vw), 1436 (w), 1130 (w), 1107 (vw), 1080 (w), 1035 (vw), 1012 (vw), 925 (vw), 862 (vw), 763 (vw), 736 (vw), 723 (vw), 694 (w), 601 (vw), 580 (vw), 563 (w), 489 (vw), 478 (vw), 460 (vw).

#### Synthesis of [Mo­(CO)_5_(PPO)­Cl_2_Lu­{μ-Cl_3_}­LuCl­(PPO)_2_(Mo­(CO)_5_)_2_] (10)

LuCl_3_ (30.1 mg, 0.11 mmol) and MoPPO (100.0 mg, 0.16 mmol) were dissolved in DCM (5 mL) and stirred for 2 h. The yellow solution was then filtered and after layering with *n*-pentane tiny pale-yellow needle like crystals of **10** suitable for X-ray diffraction were obtained. Crystalline yield: 87.0 mg, 0.04 mmol, 67%. ^1^H NMR (298 K, CD_2_Cl_2_, 400 MHz) δ [ppm] 7.80–7.70 (4H, m, P_1_(IV)–Ph–H-*ortho*), 7.70–7.48 (20H, m, P_2_(II)–Ph–H-*ortho*, P_3,5_(IV), P_4,6_(II)–Ph–H-*ortho*), 7.48–7.43 (8H, m, P_1_(IV)–Ph–H-*para*, P_1_(IV)–Ph–H-*meta*, P_2_(II)–Ph–H-*para*), 7.43–7.33 (12H, m, P_3,5_(IV)–Ph–H-*para*, P_2_(II)–Ph–H-*meta*, P_4,6_(II)–Ph–H-*para*), 7.33–7.08 (16H, m, P_3,5_(IV)–Ph–H-*meta*, P_4,6_(II)–Ph–H-*meta*). ^31^P­{^1^H} NMR (298 K, CD_2_Cl_2_, 162 MHz) δ [ppm] = 53.0 (d, P_3,5_(IV),^1^ *J*_PP_ = 147.4 Hz), 51.0 (d, P_1_(IV),^1^ *J*_PP_ = 131.7 Hz), 45.4 (d, P_4,6_(II),^1^ *J*_PP_ = 147.4 Hz), 38.2 (d, P_2_(II),^1^ *J*_PP_ = 131.7 Hz). ^13^C­{^1^H} NMR (298 K, CD_2_Cl_2_, 101 MHz) spectrum exhibited very weak signals, preventing definitive peak assignments (Figure S27). Elemental analysis, calcd for [Mo­(CO)_5_(PPO)­Cl_2_Lu­{μ-Cl_3_}­LuCl­(PPO)_2_(Mo­(CO)_5_)_2_] C 43.01, H 2.49; found C 43.96, H 2.75. ATR-IR [cm^–1^] 2077 (w), 1938 (vs), 1924 (vs), 1434 (vw), 1130 (vw), 1074 (w), 744 (vw), 690 (vw), 603 (vw), 582 (w), 559 (vw), 493 (vw), 476 (vw).

## Results and Discussion

### Monometallic Rare Earth Complexes

Consistent with the HSAB principle, the PPO/POP ligand scaffold was employed for the synthesis of both monometallic RE complexes and heterobimetallic RE/TM assemblies. Within these complexes, the hard RE­(III) centers preferentially coordinate to the oxygen donor of the PPO moiety, whereas the softer TM centers bind through the phosphorus donor sites (Figure 1). The structural outcomes of these reactions were found to depend sensitively on both the nature of the RE ion and choice of solvent.

For an initial investigation, AlCl_3_ was reacted with the PPO ligand in THF. Based on the successful formation of [AlCl_3_(PPO)] (**1**), the concept was extended toward the coordination of rare earth elements. Therefore, the respective RECl_3_ (RE = Sm, Dy, Er, Yb) and the PPO ligand was reacted in a 1:1 or 1:2 ratio, respectively, in either THF or acetonitrile for 2 h (Scheme 2). Initial reactions were performed using equimolar amounts of ligand and rare earth chloride. However, for the Sm and Dy systems the preferential formation of complexes featuring two coordinated PPO ligands per lanthanide center was consistently observed, independent of the initial metal-to-ligand ratio. The metal-to-ligand stoichiometry was therefore adjusted to increase the isolated yields of the Sm and Dy complexes. Unfortunately, further control over the number of coordinating PPO ligands through stoichiometric variations alone is not possible. After the reactions, the solutions were filtered and layered with *n*-pentane or *n*-heptane to promote crystallization. After few days, crystals of the corresponding complexes [SmCl_3_(PPO)_2_(THF)] (**2**), [DyCl_3_(PPO)_2_(CH_3_CN)] (**3**), [ErCl_3_(PPO)­(THF)_2_]·0.5 THF (**4**) and [YbCl_3_(PPO)­(THF)_2_]·0.5 THF (**5**) suitable for single crystal X-ray diffraction (SC-XRD) analysis were obtained. The crystals were colorless except for complex **4**, which had a slight pink coloration.

**Scheme 2:**
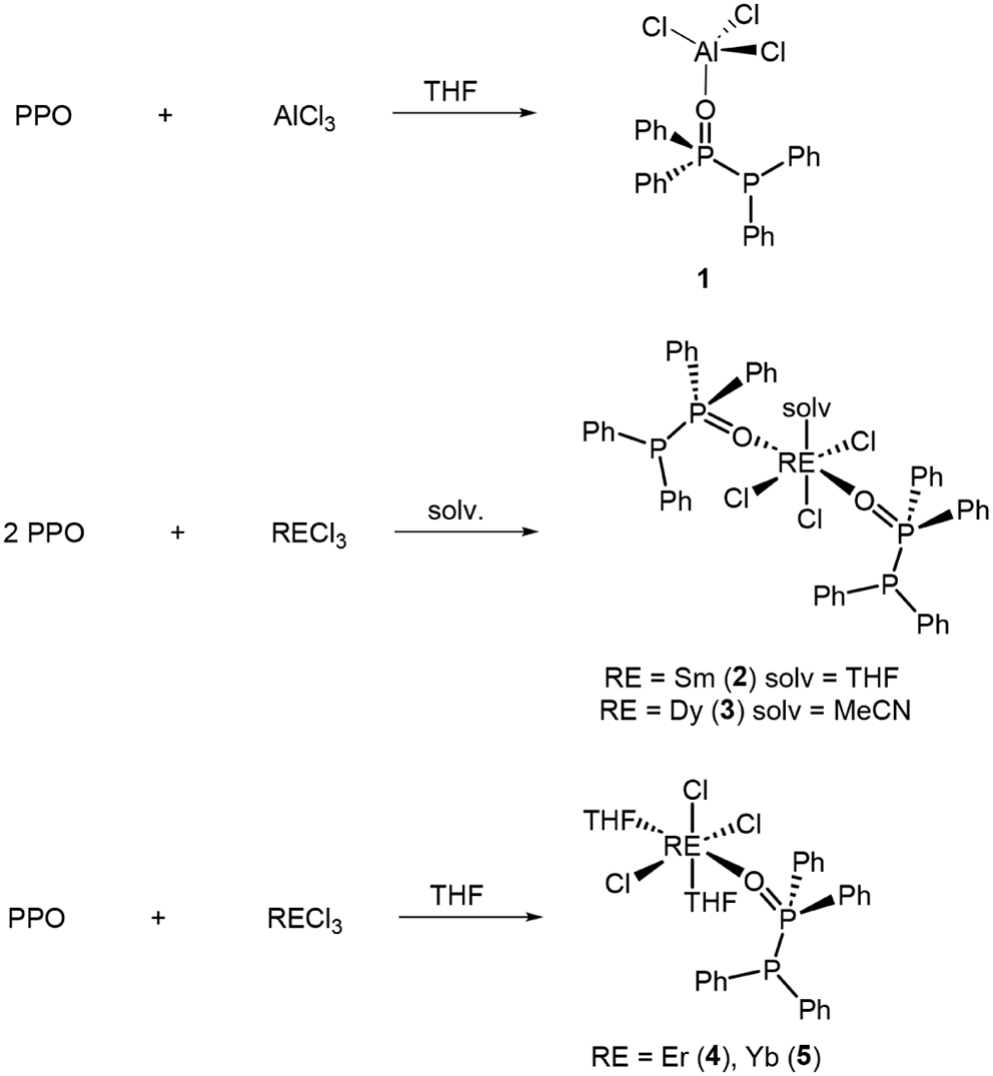
Reactions of PPO with AlCl_3_ and different RECl_3_ in various solvents affording PPO-stabilized complexes.

X-ray crystallographic analysis revealed that **2** and **3** both exhibit a 1:2 metal to ligand stoichiometry. In **2** and **3** the metal centers are coordinated by two PPO ligands *via* their oxygen atoms and three chlorine atoms (Figure 3). The ionic radii of Sm­(III) (1.079 Å) and Dy­(III) (0.912 Å) are relatively large.^61^ As a result, the rare earth ion can accommodate more than one PPO ligand without significant steric hindrance. While in **2** a THF molecule is coordinated to the Sm center, in **3** this position is occupied by an acetonitrile ligand. Consequently, the RE ions in **2** and **3** each exhibit an overall coordination number of six with an octahedral geometry.

**Figure 3:**
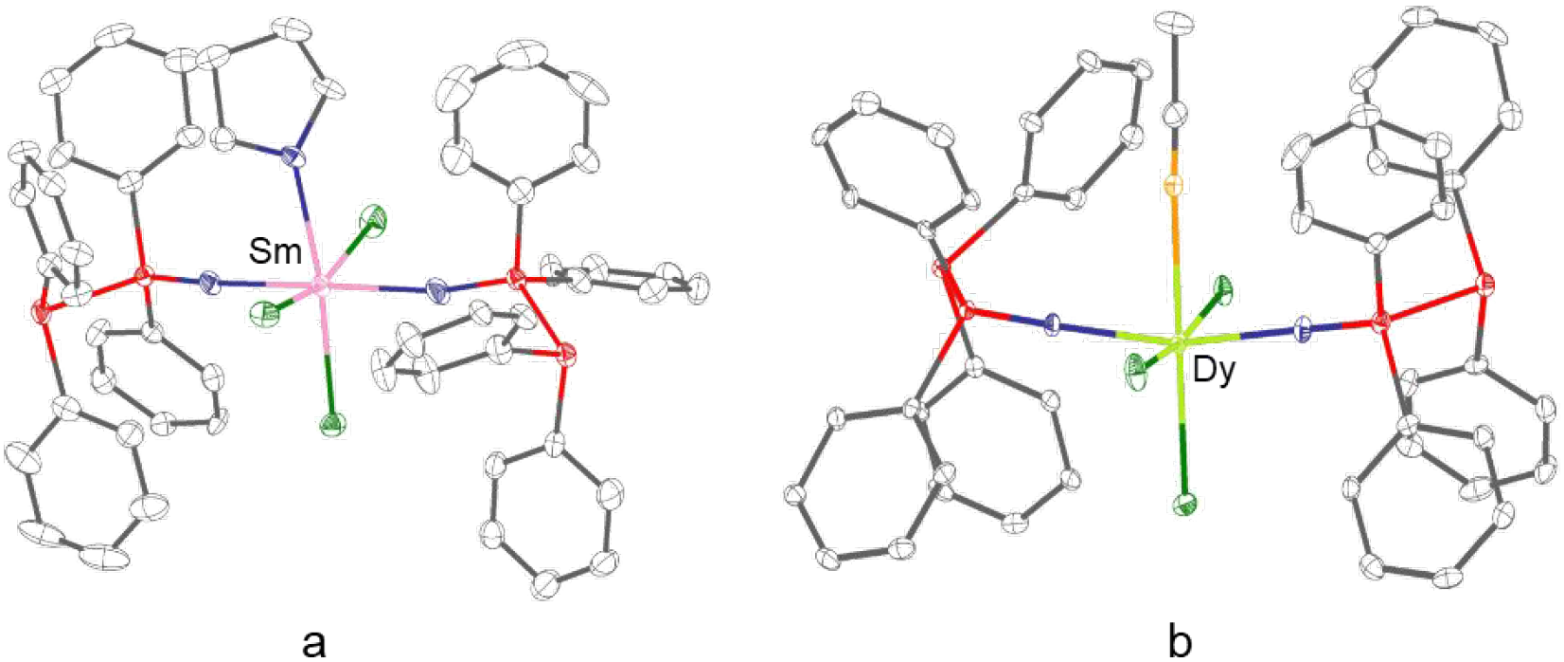
Molecular structures of a) [SmCl_3_(PPO)_2_(THF)] (**2**) and b) [DyCl_3_(MeCN)­(PPO)_2_] (**3**). Gray: carbon; blue: oxygen; red: phosphorus; green: chlorine; light orange: nitrogen.

In contrast, **1**, **4**, and **5** exhibit a 1:1 metal to ligand stoichiometry, where each metal center is coordinated by only one PPO ligand *via* the oxygen donor (Figure 4). In complex **1**, the Al is coordinated by one PPO ligand and three chloride ions, resulting in a coordination number of four. In complexes **4** and **5,** the Er and Yb centers each coordinate to one PPO ligand, two THF solvent molecules and three chloride ions, giving rise to an overall octahedral coordination geometry and a coordination number of six, as seen before in the cases of **2** and **3**.

**Figure 4:**
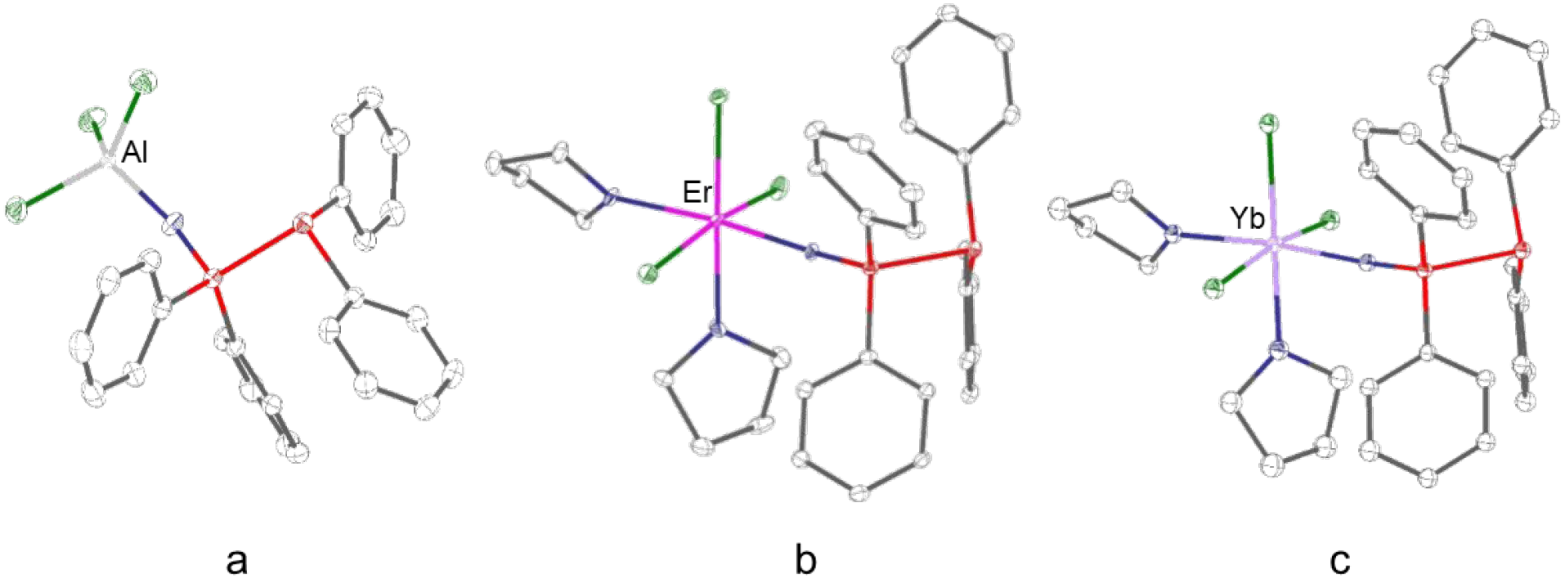
Molecular structures of a) [AlCl_3_(PPO)] (**1**), b) [ErCl_3_(PPO)­(THF)_2_] (**4**) and c) [YbCl_3_(PPO)­(THF)_2_] (**5**). Gray: carbon; blue: oxygen; red: phosphorus; green: chlorine.

Interestingly, when the solvent of the reaction was changed from THF or acetonitrile to DCM, a completely different structure was obtained when using the yttrium precursor. In this case a dinuclear complex of the form [(PPO)­Cl_2_Y­{μ-Cl_3_}­YCl­(PPO)_2_] (**6**), was obtained, which will be discussed later.

The reason for the different coordination environments for **2** and **3** compared to **1**, **4** and **5** is most likely caused by the increased ionic radii of Sm­(III) and Dy­(III) compared to Al­(III), Er­(III) and Yb­(III).^61^ The smaller ionic radii of Er­(III) (0.890 Å) and Yb­(III) (0.868) most likely lead to an increased steric crowding, restricting the coordination to a single PPO ligand. A similar structural arrangement was observed in case of [YCl_3_(PPO)­(THF)_2_].^45^

Comparison of the molecular structures of **1**–**5** reveals comparable P–O bond lengths, ranging from 1.508(3) Å to 1.519(6) Å (Table 1). These bond lengths are slightly longer than the P–O bond length in the free PPO ligand,^62^ consistent with oxygen coordination to the Al/RE centers. The variations among the RE complexes correlate with differences in ionic radius and Lewis acidity of the RE: the larger, less Lewis-acidic Sm­(III) and Dy­(III) ions form weaker M–O interactions than the smaller, harder Er­(III), Yb­(III), and Al­(III) ions.^61^ Consequently, harder metals exhibit shorter M–O bonds. In particular, the Al–O distances (complex **1**) are significantly shorter, reflecting its higher charge density and greater covalent character, which in turn slightly lengthens the P–O bond. The P–P bond lengths (2.180(3) Å–2.206(16) Å) in **1**–**5** are marginally shorter than that of the free PPO ligand (2.213(6) Å).^62^ The O–RE–O bond angle (RE = Sm, Dy) differs by about 9° between complexes **2** and **3**, likely due to steric effects. The coordination of THF in **2** widens this angle through interaction with the adjacent phenyl rings, whereas the linear acetonitrile ligand in **3** minimizes steric hindrance, yielding a smaller O–RE–O angle (Table 1).

**Table 1:** Selected bond lengths and bond angles of the monometallic PPO complexes **1**–**5.** ^a^

	**1**	**2**	**3**	**4**	**5**
P–O (Å)	1.539(2)	1.512(6), 1.520(6)	1.511(3), 1.511(3)	1.514(3)	1.508(3)
Al/RE–O (Å)	1.762(2)	2.316(6), 2.336(6)	2.224(3), 2.251(3)	2.211(2)	2.197(3)
P–P (Å)	2.203(8)	2.200(3), 2.180(3)	2.1983(18), 2.2024(18)	2.2009(15)	2.2059(16)
P–O–RE (°)	142.7(13)	170.6(4), 167.6(4)	171.3(2), 178.2(3)	169.65(17)	169.2(2)
O–RE–O (°)		174.4 (2)	165.87(12)		
O–P–P (°)	105.8(8)	115.4(3), 119.1(3)	118.35(15), 117.11(14)	115.84(12)	116.03(14)

a ^31^P­{^1^H} NMR spectroscopy supports the coordination of the RE metals to the ligand *via*oxygen. The free PPO ligand shows two doublets at 35.8 and −21.9 ppm (^1^ *J*_PP_ = 227.7 Hz, CDCl_3_), corresponding to P­(=O) and PPh_2_.^45^ Upon complexation, the P­(IV) signal shifts to 50 ppm (**1**) and 45 ppm (**2**) with pronounced broadening, which confirms the metal–oxygen coordination. The deshielding reflects a decrease in electron density at the phosphorus atom due to metal binding. For Dy­(III), Er­(III), and Yb­(III) complexes, paramagnetism leads to broad ^31^P­{^1^H} NMR spectra, which limits further NMR spectroscopic analysis.

### Heterobimetallic RE/Mo Complexes

Initial attempts to introduce a soft transition metal to the vacant soft phosphorus donor atom of the PPO ligand in the monometallic RE complexes has proven to be challenging. When Cu­(I) or Au­(I) salts were reacted with **2**, **3**, **4** or even [YCl_3_(PPO)­(THF)_2_],^45^ the desired heterobimetallic complexes could not be isolated, although NMR spectroscopic studies indicated coordination for example in the case of Cu­(I) (Figure S28). This lack of success was attributed to the propensity of Cu­(I) and Au­(I) to promote the isomerization of the PPO to the POP tautomer, which precludes the coordination of the RE metal by the oxygen atom and only results in the isolation of pure TM-containing POP-type complexes.^46,63,64^ Interestingly, when **2** was reacted with [Cu­(CH_3_CN)_4_]­PF_6_ in THF, the ring-opening polymerization of tetrahydrofuran was observed. The polymerization activity could be attributed to heterobimetallic metal cooperativity, whereby the rare earth ions assist in the preorganization and activation of the THF monomers, whereas Cu­(I) plays a key role in the tertiary oxonium ion formation. A comprehensive compound screening has shown that [Cu­(CH_3_CN)_4_]­PF_6_ and RECl_3_ (RE = La, Ce, Nd, Sm), even without the presence of an organic ligand, enable the controlled, ring-opening polymerization of THF and the formation of high-molecular-weight poly-THF. Notably, neither the rare earth nor the copper species was catalytically active on its own.^65^

A precedent from 1982 offered a viable strategy: the isolation of a stable Mo(0) complex, in which the PPO tautomer coordinates through its phosphorus atom, [(Mo­(CO)_5_(PPO)].^51^ This complex will be abbreviated as MoPPO in the following. This remains one of the very few documented cases in which the PPO tautomer binds a transition metal *via* its soft P-donor without undergoing isomerization to the more common POP form, typically favored by soft metal centers.^66–68^ Notably, in MoPPO the PPO tautomer preserves a free coordination site at the oxygen atom, making it particularly attractive for subsequent heterobimetallic complex formation. Based on this, we changed our strategy, isolating the Mo-based complex first and then using this as a synthon for the subsequent coordination of the RE. We have therefore reacted MoPPO with RECl_3_ (RE­(III) = Y, Dy, Lu) in THF (Scheme 3). Subsequent *n*-pentane diffusion into the reaction mixtures afforded crystalline products. Notably, the reaction of the homometallic RE–PPO complexes with [Mo­(CO)_5_] did not result in any product formation.

**Scheme 3:**
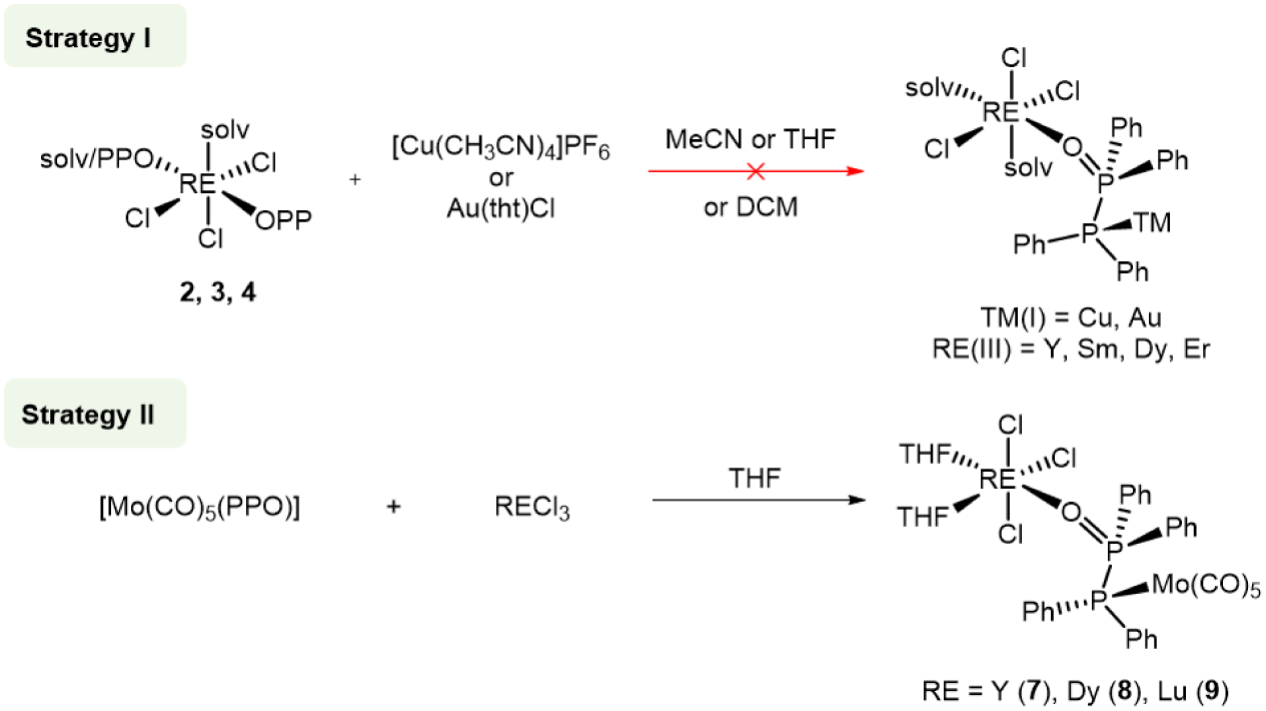
Strategies for the synthesis of heterobimetallic RE/TM complexes using the PPO/POP ligand. tht = tetrahydrothiophene

X-ray crystallographic analysis revealed the heterobimetallic complexes **7**–**9** with a 1:1:1 RE to Mo to ligand ratio. The structures show that each RE­(III) (RE = Y, Dy and Lu) center is coordinated by the oxygen atom of the PPO ligand, three chloride ions, and two THF molecules, resulting in a coordination number of six and an overall octahedral coordination geometry. On the other side of the ligand, the molybdenum center is coordinated to the phosphorus atom of the PPO ligand and five carbonyl groups (Figure 5).

**Figure 5:**
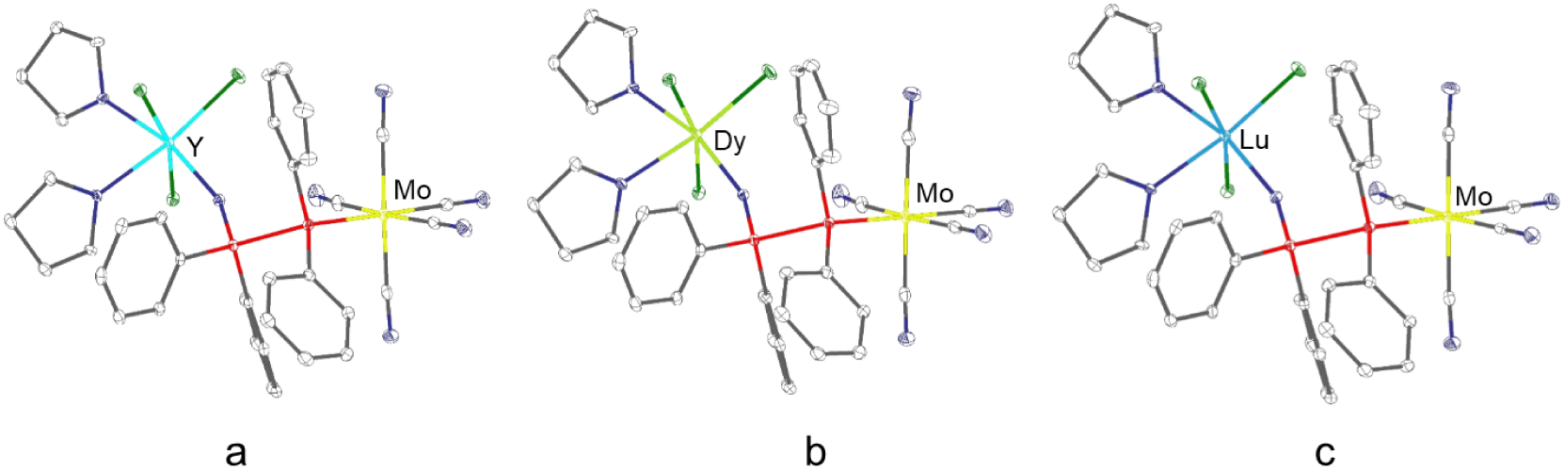
Molecular structures of a) [YCl_3_(PPO)­(THF)_2_Mo­(CO)_5_] (**7**), b) [DyCl_3_(PPO)­(THF)_2_Mo­(CO)_5_] (**8**) and c) [LuCl_3_(PPO)­(THF)_2_Mo­(CO)_5_] (**9**). Gray: carbon; blue: oxygen; red: phosphorus; green: chlorine.

In comparison to the monometallic RE complexes, the heterobimetallic RE/Mo complexes showed only minor changes in the P–O and RE–O bond lengths, while a significant P–P bond elongation was observed (Table 2). This elongation likely arises because of the dual coordination of Mo(0) by the P­(II) atom and RE­(III) by the oxygen atom, which results in a shift of the electron density toward the metal centers. This electron density shift also induces a bend in the ligand backbone. As a result, the P–O–RE angles decrease by roughly 15°, compared to the monometallic analogues.

**Table 2:** Selected bond lengths and bond angles of the PPO-based complexes **6**–**10.**

	**6**	**7**	**8**	**9**	**10**
P–O (Å)	1.519(4), 1.526(5), 1.523(4)	1.515(17)	1.516(3)	1.512(3)	1.520(3), 1.512(3), 1.513(3)
RE–O (Å)	2.207(4), 2.195(5), 2.186(3)	2.227(15)	2.243(2)	2.193(2)	2.129(3), 2.171(3), 2.161(3)
P–P (Å)	2.197(19), 2.199(19), 2.184(18)	2.246(6)	2.243(9)	2.246(11)	2.226(2), 2.248(17), 2.256(19)
Mo–P (Å)		2.529(6)	2.529(9)	2.528(10)	2.512(19), 2.524(13), 2.529(13)
Mo–CO (Å)		2.007(2)–2.070(2)	2.007(3)–2.070(4)	2.005(3)–2.075(4)	1.984(6)–2.089(8)
P–O–RE (°)	172.6(3), 164.2(2), 172.9(2)	157.94(9)	157.84(14)	157.94(14)	156.4(2), 164.1(2), 167.04(19)
O–P–P (°)	117.9(15), 117.7(16), 117.8(15)	107.08(6)	107.19(9)	107.47(9)	110.86(14), 108.49(14), 112.10(12)
Mo–P–P (°)		112.30(3)	112.25(4)	112.57(4)	113.06(6), 113.26(7), 118.62(7)

To gain further insights into the coordination behavior of the heterobimetallic complexes, ^31^P­{^1^H} NMR spectroscopy was conducted for the diamagnetic complexes **7** and **9** (Figure S27). In comparison, the spectrum of the monometallic MoPPO complex in CDCl_3_ displays two doublets at 34.5 and 32.2 ppm (^1^ *J*_PP_ = 107.0 Hz). In coordinating solvents, such as THF-*d*_8_ or CD_3_CN, the spectra of complexes **7** and **9** show signals corresponding to the intact heterobimetallic complexes and homometallic RE–PPO complexes, resulting from the dissociation of the Mo unit. This becomes evident from a comparison of the ^31^P­{^1^H} NMR spectrum of **7** in THF-*d*_8_ and the literature reported [YCl_3_(THF)_2_(PPO)] complex, which exhibits signals at 51 and −20 ppm.^45^ In other solvents, such as CDCl_3_ and CD_2_Cl_2_, complex multiplets are observed, which can indicate the decomposition of the complexes.

### Solvent Effect and Formation of Dinuclear Complexes

When coordinating solvents (e.g., THF or acetonitrile) were replaced with the noncoordinating solvent dichloromethane (DCM), the reactions afforded homometallic RE and heterobimetallic RE/Mo complexes (**6** and **10**), both featuring a RE_2_Cl_3_ core (Scheme 4). This solvent-dependent structural divergence between the RE and RE_2_ cores likely arises from differences in the coordinating ability of the solvents. Such variations can influence both the solubilization of the rare earth chlorides and the stability of the mononuclear RE–PPO complexes.

**Scheme 4:**
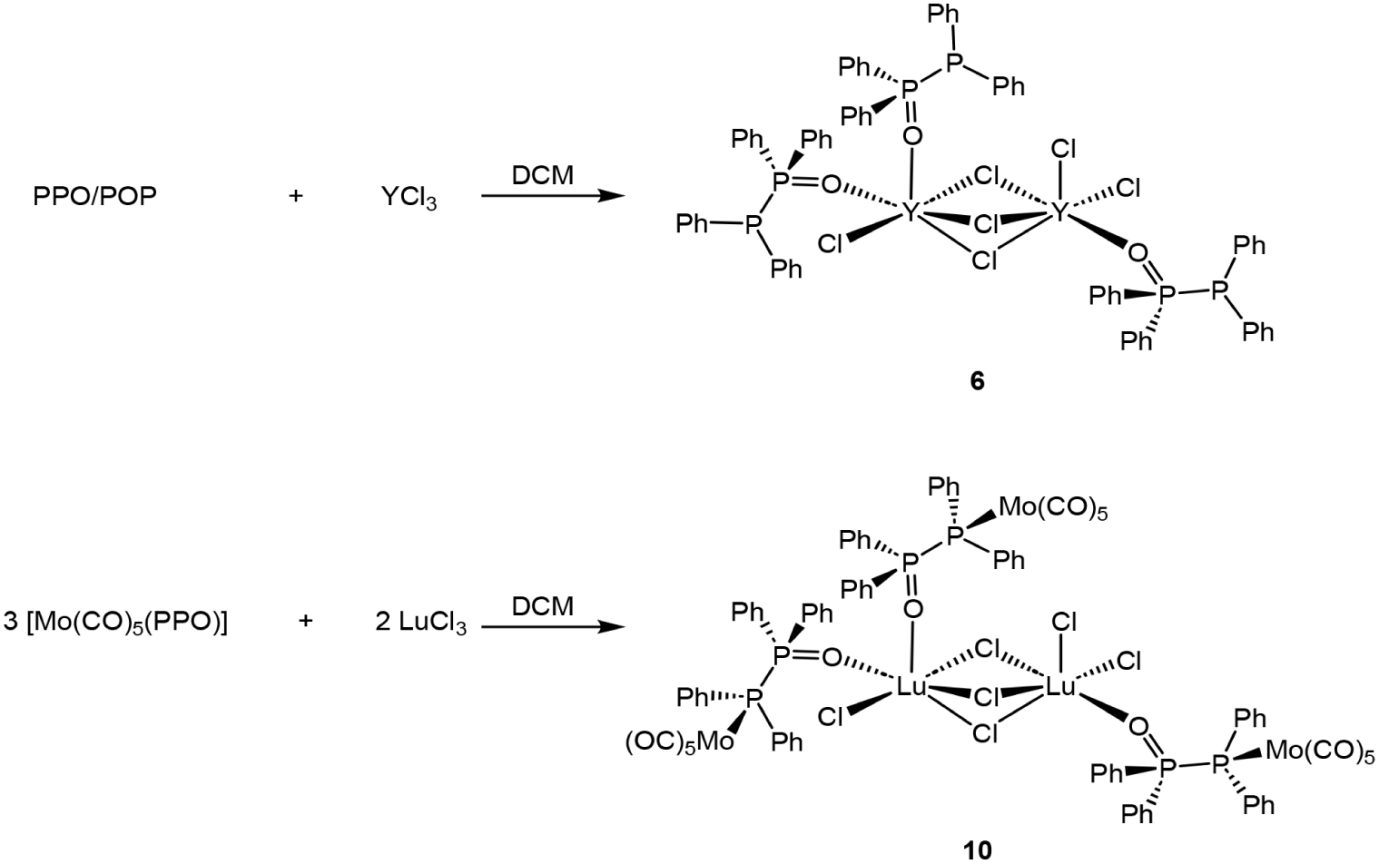
Reaction of PPO/POP with YCl_3_ and reaction of MoPPO with LuCl_3_ in DCM forming chlorine-bridged dinuclear RE cores.

The X-ray crystallographic analysis shows that these complexes adopt a 2:3 stoichiometry of RE metal to ligand. Their molecular structures feature asymmetric coordination environments, in which one RE center is bound to a single PPO ligand and two chloride ions, whereas the second RE center is coordinated by two PPO ligands and one chloride ion (Figure 6). The two RE atoms are interconnected through three bridging chloride ions, resulting in a dinuclear core in which each RE center has the coordination number six. In complex **6**, the distance between the two Y­(III) centers is 3.73 Å and in **10**, the Lu­(III) centers are 3.63 Å apart.

**Figure 6:**
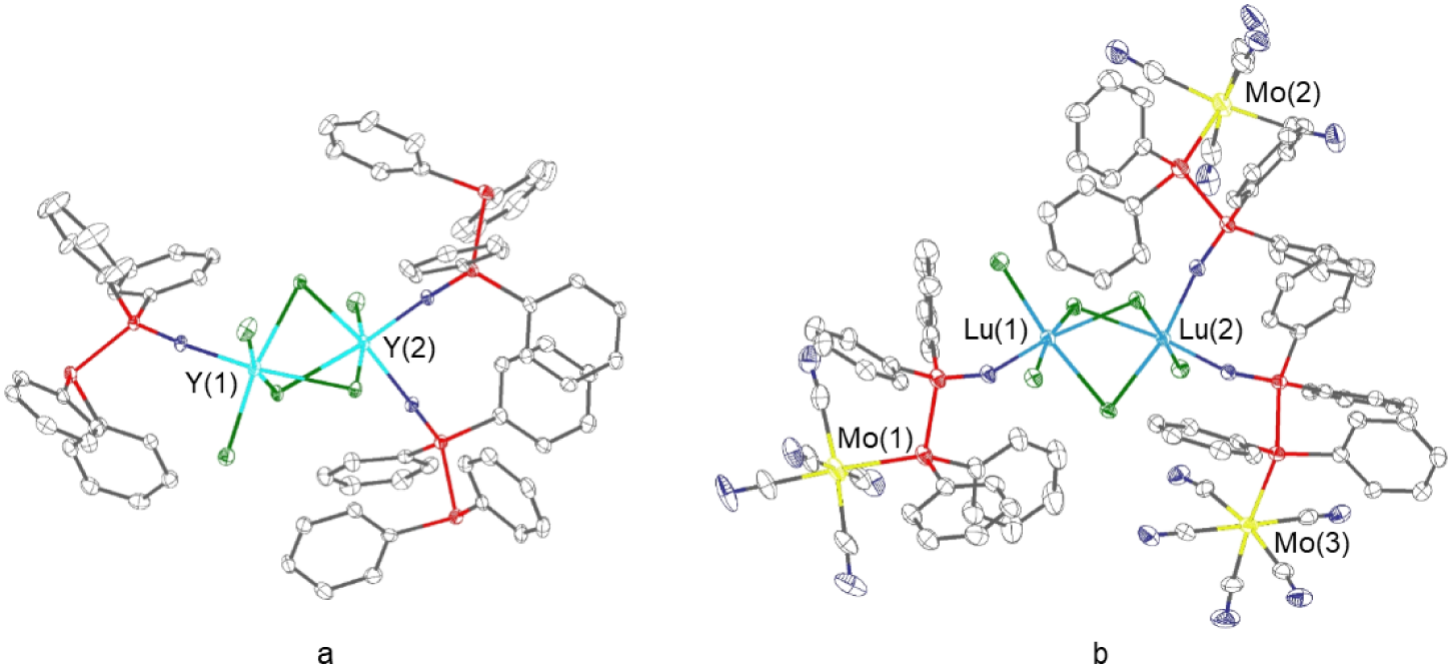
Molecular structures of a) [(PPO)­Cl_2_Y­{μ-Cl_3_}­YCl­(PPO)_2_] (**6**) and b) [Mo­(CO)_5_(PPO)­Cl_2_Lu­{μ-Cl_3_}­LuCl­(PPO)_2_Mo­(CO)_5_] (**10**). Hydrogen atoms have been excluded for clarity, and ellipsoids are depicted at the 30% probability level. Gray: carbon; blue: oxygen; red: phosphorus; green: chlorine.

An unusual intermolecular interaction between the two adjacent carbonyl oxygen atoms from neighboring Mo–CO units was observed in complex **10** (Figure S42). The O···O distance of 2.71 Å is significantly shorter than that of typical van der Waals separations, suggesting a weak attractive interaction, which most likely arises from dense crystal packing. Such O···O contacts are exceedingly rare and, to the best of our knowledge, have not been previously reported in molybdenum carbonyl or related transition-metal carbonyl systems.^69^

Further insights into the solution stability of complexes **6** and **10** was obtained from NMR spectroscopy. Whereas the complexes **7** and **9** show decomposition and rearrangement reactions in solution, the formation of the RE_2_Cl_3_ cores in **6** and **10** seem to enhance the solution stability substantially. The ^31^P­{^1^H} NMR spectrum of **6** in CD_2_Cl_2_ reveals four doublets, two of which show twice the intensity of the others (Figure 7). The weaker doublets at −18.6 and 51.0 ppm (^1^ *J*_PP_ = 284.8 Hz) correspond to a single PPO ligand coordinated to one yttrium center, whereas the more intense signals at −16.1 and 52.0 ppm (^1^ *J*_PP_ = 284.8 Hz) arise from two PPO ligands bound to a second yttrium site in an equivalent environment. A pronounced downfield shift of the P­(IV) resonance from 35.8 ppm in the free ligand (CDCl_3_) to 51.0–52.0 ppm confirms the coordination of the PPO-oxygen to yttrium. Similarly, the P­(II) resonance shifts upfield from −22.5 ppm to −16.1 and −18.6 ppm, accompanied by a doublet of doublets (^2^ *J*_PY_ = 10.6 Hz) for P­(IV), consistent with coupling to the NMR-active ^89^Y nucleus (I = 1/2, 100% abundance).

**Figure 7:**
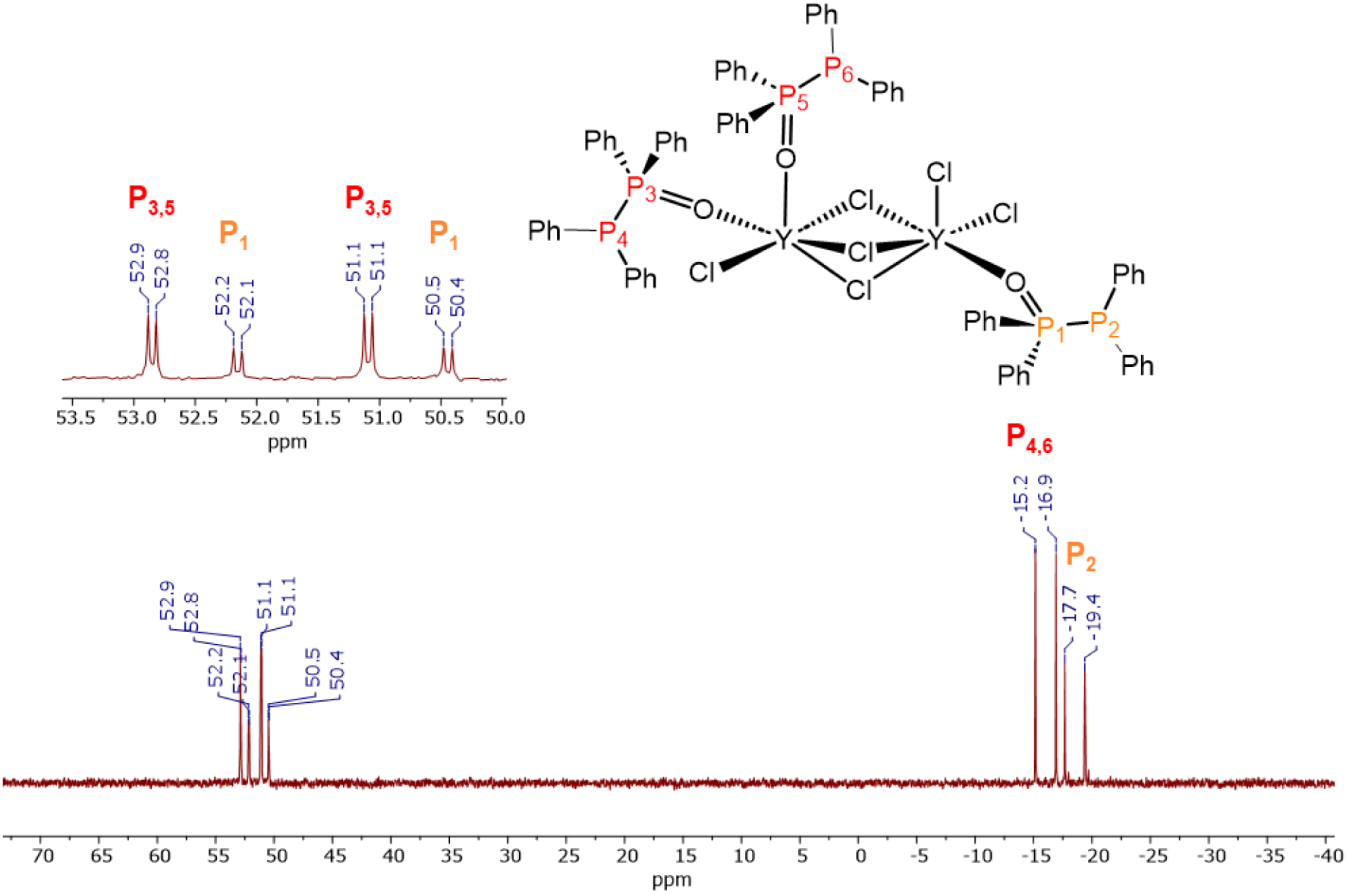
^31^P­{^1^H} NMR spectrum of **6** in CD_2_Cl_2_, indicating the arrangement of the PPO ligand in the complex.

^31^P­{^1^H} NMR spectroscopic analysis of complex **10** revealed downfield shifts of the P­(IV) signals, indicating the coordination of the oxygen atoms of the PPO ligands to Lu­(III) (Figure S30). Furthermore, the P­(II) resonances in **10** appear significantly downfield at 45.4 ppm (^1^ *J*_PP_ = 148.9 Hz) and 38.2 ppm (^1^ *J*_PP_ = 131.9 Hz), compared to 32.2 ppm (^1^ *J*_PP_ = 107.0 Hz) in the MoPPO complex, reflecting the withdrawal of electron density upon Lu coordination. The observed variations in the P­(II) chemical shifts between the heterobimetallic Lu/Mo–PPO and monometallic Mo–PPO complexes suggest that the RE coordination alters the electronic environment of the P­(II) atom and, in turn, potentially affects the Mo center, even though it is separated significantly from the rare-earth ion.

### Further Spectroscopic Analysis

As discussed earlier, the PPO ligand exists in a phosphorotropic equilibrium with its POP tautomer, which was proven by single crystal structural analysis and ^31^P­{^1^H} NMR spectroscopy.^45^ Fourier-Transform Infrared spectroscopy (FT-IR) analyses further supports this assignment, showing the characteristic P=O stretching vibration, whose position varies with the nature of the coordinating metal (Table 3). In the free PPO ligand, the ν­(P=O) band appears at 1175 cm^–1^. Upon coordination to RE ions, this band undergoes a redshift, indicating a decrease in P=O bond order as a result of metal coordination (Figure 8). The magnitude of this red shift correlates with the Lewis acidity and coordination environment of the metal centers. Interestingly, complex **1** shows the lowest wavenumber (1114 cm^–1^) among all complexes, which is in line with significant electron density withdrawal by the highly Lewis-acidic Al­(III) center. The subsequent coordination by the P­(II) atom in the PPO ligand attenuates the P=O stretching frequencies in the heterobimetallic complexes **7**–**10**, likely due to electron withdrawal by the Mo center through the P–P bridge, which reduces the P=O bond polarity.

**Table 3:** Characteristic absorption wavelengths of MoPPO and complexes **1**–**10.**

	ν (P=O) in cm^–1^		ν (C≡O) in cm^–1^
**1**	1114 (s)	[Mo(CO)_5_(PPO)]	1936 (vs) and 1917 (vs)
**2**	1126 (vs)	**7**	1936 (vs) and 1917 (vs)
**3**	1130 (vs)	**8**	1934 (vs) and 1917 (vs)
**4**	1130 (vs)	**9**	1953 (vs) and 1926 (vs)
**5**	1137 (vs)	**10**	1938 (vs) and 1924 (vs)
**6**	1118 (s)		

**Figure 8:**
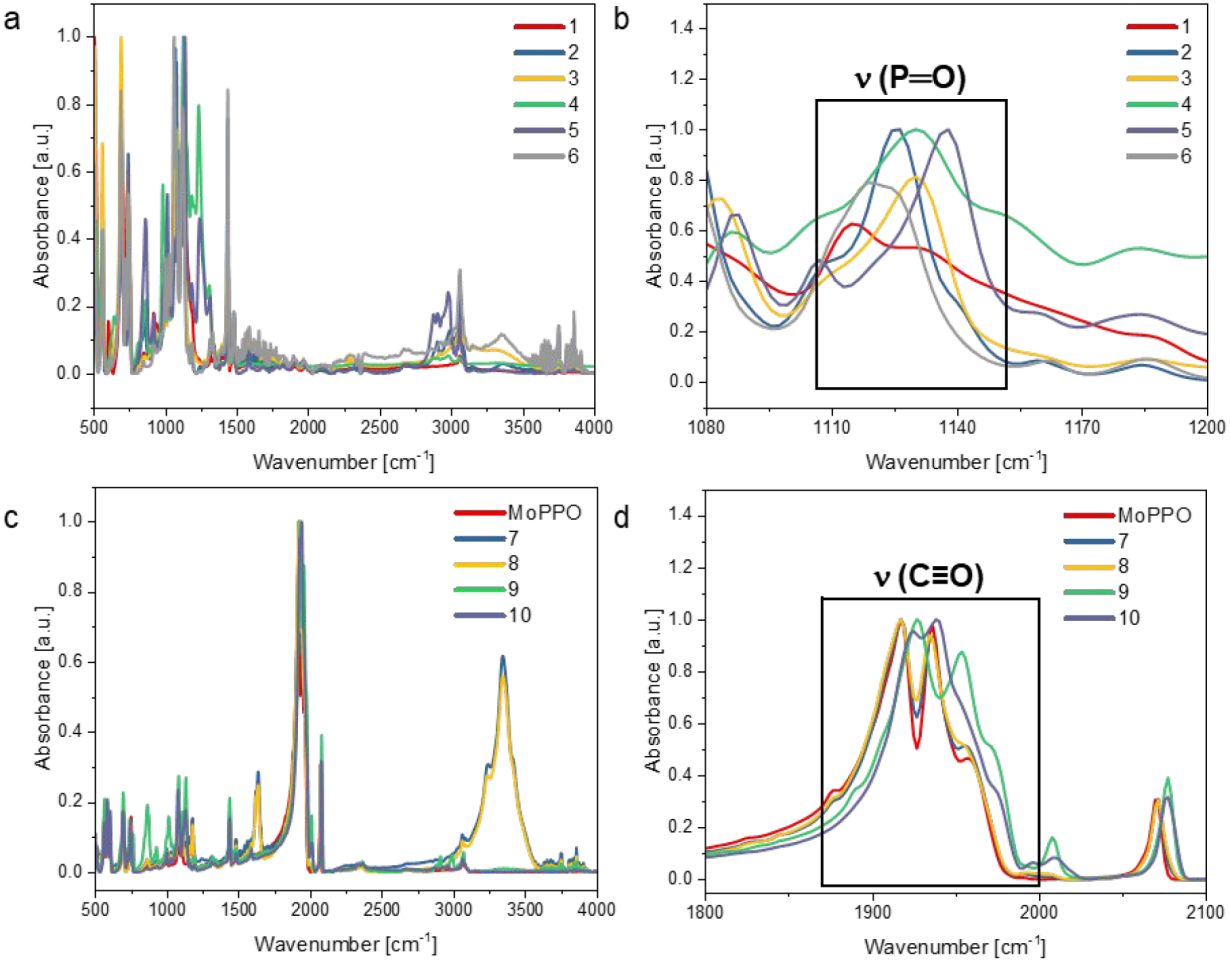
FT-IR spectra of the a) monometallic Al/RE complexes (**1**–**6**) and c) the heterobimetallic RE/Mo complexes (**7**–**10**). Inserts into the relevant regions of the b) P=O and d) C≡O bands.

In addition to the P=O stretching vibration, the C≡O stretching frequencies of the heterobimetallic RE/Mo complexes provide valuable information about their electronic structures and possible metal–metal interactions. The Lu complexes **9** and **10** show pronounced blueshifts of the C≡O bands compared to MoPPO. Because the Lu–Mo distances in these complexes are large, direct metal–metal interactions can be excluded. Instead, coordination of the strongly Lewis-acidic Lu^3+^ centers by the PPO oxygen atoms withdraws electron density from the ligand framework. This electron withdrawal reduces the P→Mo donation, which in turn diminishes Mo→CO π-backbonding. The resulting decrease in electron density at the Mo center accounts for the observed blueshift of the carbonyl stretching frequencies. No comparable electronic perturbation is observed in the C≡O stretching frequencies of the Y (**7**) and Dy (**8**) complexes, which may be attributed to the greater Lewis acidity of Lu relative to Y and Dy.

The electronic interactions suggested by the IR spectral shifts are further supported by the UV–Vis spectra, which demonstrate how coordination of the Lewis-acidic RE ions modulates the electronic transitions of the MoPPO unit. The solution UV–Vis spectra of complexes **7**–**10** show a series of absorptions in the range of 250–475 nm, with intense absorptions in the region 390–420 nm (ε = 78–1506 L·cm^1^·mol^–1^, ESI Table S1, Figure S41). The PPO ligand has no absorption in this region, while the monometallic MoPPO displays a band at 409 nm (ε = 78 L·cm^1^·mol^–1^). Given the lack of any corresponding absorption from the free ligand and the modest molar absorptivity, this feature is tentatively assigned to a weakly allowed metal-to-ligand charge-transfer (MLCT) transition, in agreement with other reported Mo-CO complexes.^70^ All heterobimetallic complexes show similar λ_max_ values, indicating that the coordination with Lewis acidic RE ions does not significantly alter the transition energy, but notably increases the molar extinction coefficient. This effect is likely caused by an increase in the transition dipole moment and enhanced ligand polarization induced by coordination to the RE­(III) center. Complexes **7** and **9** exhibit comparable ε (407 and 433 L·cm^1^·mol^–1^) consistent with the similar Lewis acidity of Y­(III) and Lu­(III), whereas complex **8**, incorporating the less Lewis acidic Dy­(III), shows a lower ε (301 L·cm^1^·mol^–1^). In complex **10**, which contains two Lu centers bridged by three chlorides and three ligands, extended charge transfer interactions result in the highest extinction coefficient (1056 L·cm^1^·mol^–1^) among the series.

## Conclusion

This work reports a systematic study on the synthesis of monometallic RE and heterobimetallic RE/Mo complexes guided by a targeted ligand design strategy. The hybrid PPO/POP ligand selected in accordance with the HSAB principle, effectively directs coordination through its bifunctional hard oxygen and soft phosphorus donor sites. The hard RE ions preferentially binds to the oxygen center, while the softer TM coordinates to the phosphorus site. Initial efforts to obtain the RE/TM based heterobimetallic complexes by incorporating soft TM precursors of Cu­(I) or Au­(I) onto the phosphorus site of the preformed RE monometallic complexes were unsuccessful. As an alternative strategy, a suitable TM synthon, MoPPO was reacted with RECl_3_. This resulted in the successful synthesis of heterobimetallic RE/Mo complexes. Furthermore, the coordination behavior of the RE metals toward PPO was found to be solvent-dependent. In the noncoordinating solvent, DCM, chloride bridged RE_2_ complexes were obtained, unlike the coordinating solvents like THF or acetonitrile that yielded mononuclear complexes. The X-ray crystallographic analysis of all the RE complexes revealed a coordination number of six of the RE ions. Despite the absence of a direct RE–Mo bond and being distantly apart from each other, the electronic interactions through the bridging PPO ligand became evident from NMR, IR and UV–Vis spectroscopy. Overall, this study establishes that targeted design of hybrid ligands is a versatile and effective strategy for constructing heterobimetallic RE/TM complexes. This methodology opens new avenues to explore their unique electronic properties and synergistic effects of rare earth and transition metals, with investigations into their catalytic applications, especially in the context of hydrofunctionalization reactions, currently underway.

## Supplementary Material























## Data Availability

The data underlying this study are openly available under DOI: 10.35097/e7jz9z18d52t5y36. We have also published a preprint in ChemRxiv (DOI: 10.26434/chemrxiv-2025-zw6m4).

## Notes

Representative NMR spectra, IR spectra, UV–vis spectra, single-crystal X-ray crystallographic details (PDF)
(CIF)
(CIF)
(CIF)
(CIF)
(CIF)
(CIF)
(CIF)
(CIF)
(CIF)
(CIF)
