# Supplementary material for: Coordination Chemistry at the Hard–Soft Interface: Phosphine Oxide-Based Rare Earth/Transition Metal Complexes
Source: ACS Omega. 2026 Feb 22;11(9):15462–75. doi: 10.1021/acsomega.5c13312 (PMC12980413; doi:10.1021/acsomega.5c13312)
Supplement: Supplementary file 1 [file ao5c13312_si_001.pdf]

Supporting Information

Coordination Chemistry at the Hard–Soft Interface: Phosphine Oxide-Based Rare Earth/Transition Metal Complexes

Rwitabrita Panda,[a], Franziska Flecken,[a], Christina Papke [a] Christopher E. Anson,[a] Toni Grell[b] and Schirin Hanf*[a]

[a] Institute for Inorganic Chemistry, Karlsruhe Institute of Technology, Engesserstr. 15,

76131 Karlsruhe, Germany, Mail. schirin.hanf@kit.edu;

[b] Dipartimento di Chimica, Institution Università degli Studi diMilano, Via Camillo Golgi

19, Milan 20131, Italy

All supporting data, in open file formats, can be found under DOI: 10.35097/e7jz9z18d52t5y36.

Contents

1. Representative NMR spectra 2
2. IR spectra 11
3. UV-Visible Spectra 16
4. Single-crystal X-ray crystallography 17

1. Representative NMR spectra

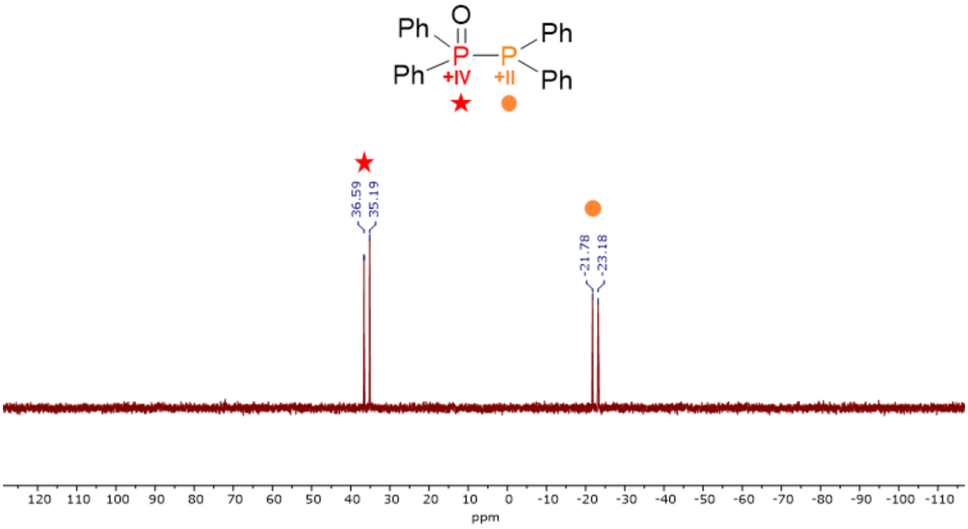

Figure S 1. $^{31}P\{^{1}H\}$ NMR (298 K, $CDCl_3$, 162 MHz) spectrum of the free PPO ligand, illustrating the oxidation states of the respective phosphorous atoms and their corresponding chemical shifts.

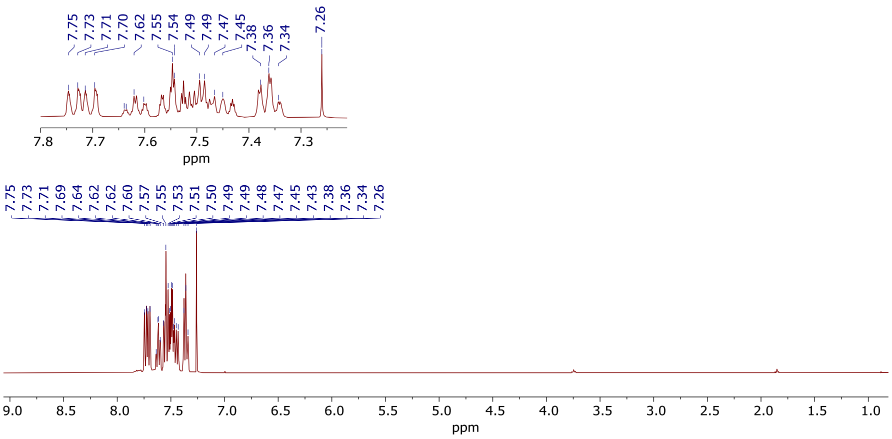

Figure S 2. ^{1}H NMR (298 K, $CDCl_3$, 400 MHz) spectrum of [$AlCl_3$(PPO)] (**1**). Note: a small amount of the solvent residual peaks of THF are visible.

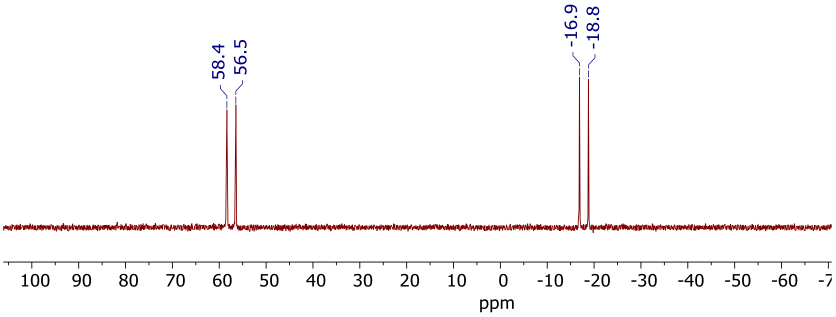

Figure S 3. $^{31}P\{^{1}H\}$ NMR (298 K, $CDCl_3$, 162 MHz) spectrum of [$AlCl_3$(PPO)] (**1**).

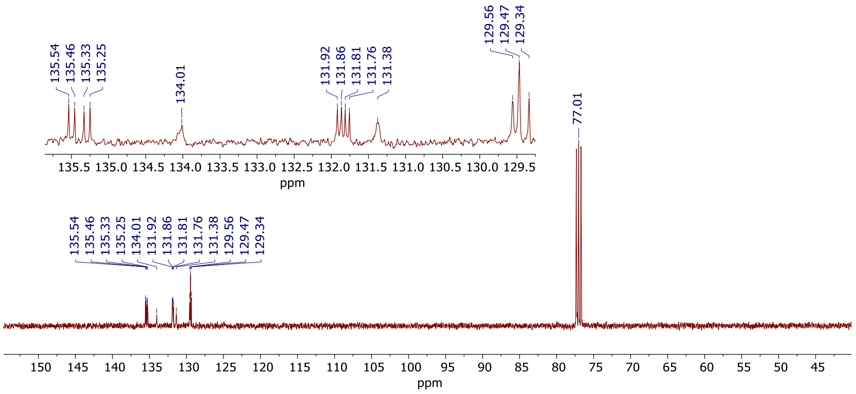

Figure S 4. $^{13}C\{^{1}H\}$ NMR (298 K, $CDCl_3$, 101 MHz) spectrum of $[AlCl_3(PPO)]$ (**1**).

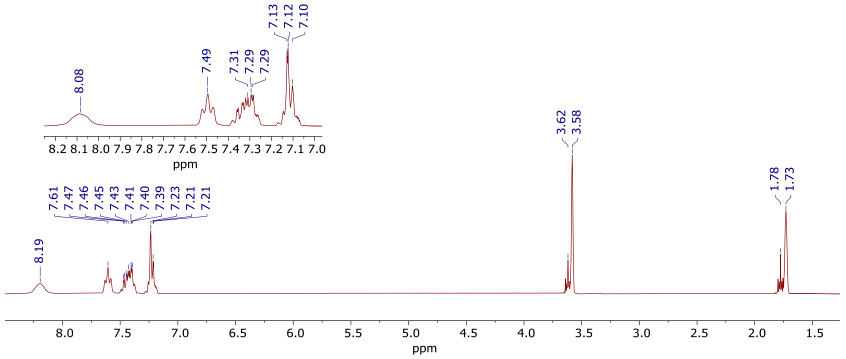

Figure S 5. ^{1}H NMR (298 K, THF-d_8, 400 MHz) spectrum of $[SmCl_3(PPO)_2(THF)]$ (**2**).

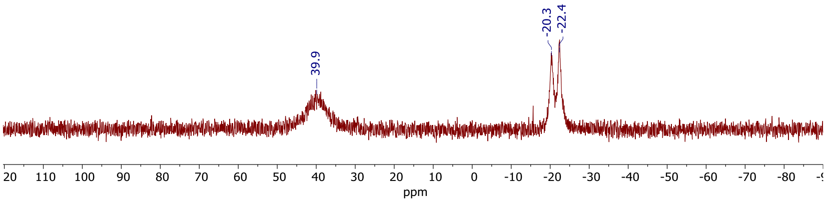

Figure S 6. $^{31}P\{^{1}H\}$ NMR (298 K, THF-d_8, 162 MHz) spectrum of $[SmCl_3(PPO)_2(THF)]$ (**2**).

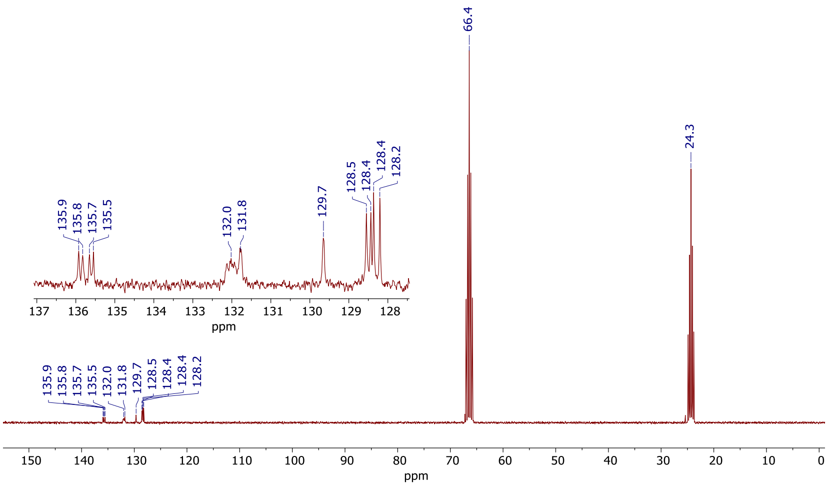

Figure S 7. $^{13}C\{^{1}H\}$ NMR (298 K, THF-d_8, 101 MHz) spectrum of $[SmCl_3(PPO)_2(THF)]$ (**2**).

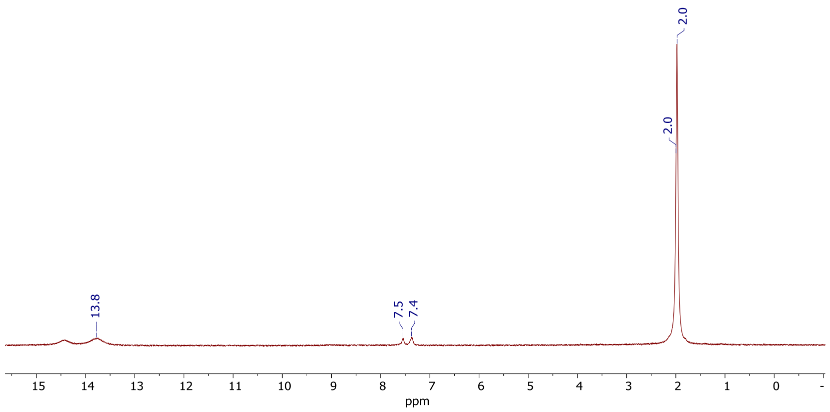

Figure S 8. ^{1}H NMR (298 K, CD_3CN, 400 MHz) spectrum of [$DyCl_3(CH_3CN)(PPO)_2$] (**3**). Note: NMR data could not be obtained due to the paramagnetic nature of the Dy(III) centre.

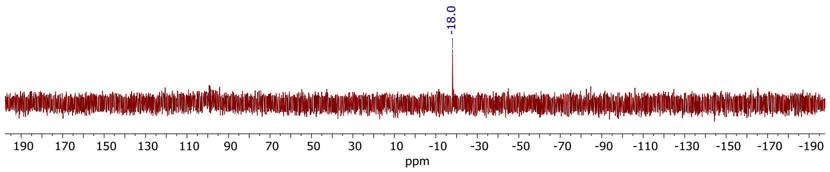

Figure S 9. ^{31}P{^{1}H} NMR (298 K, CD_3CN, 162 MHz) spectrum of [$DyCl_3(CH_3CN)(PPO)_2$] (**3**). Note: poor quality of NMR corresponds to the paramagnetic nature of the Dy(III) centre.

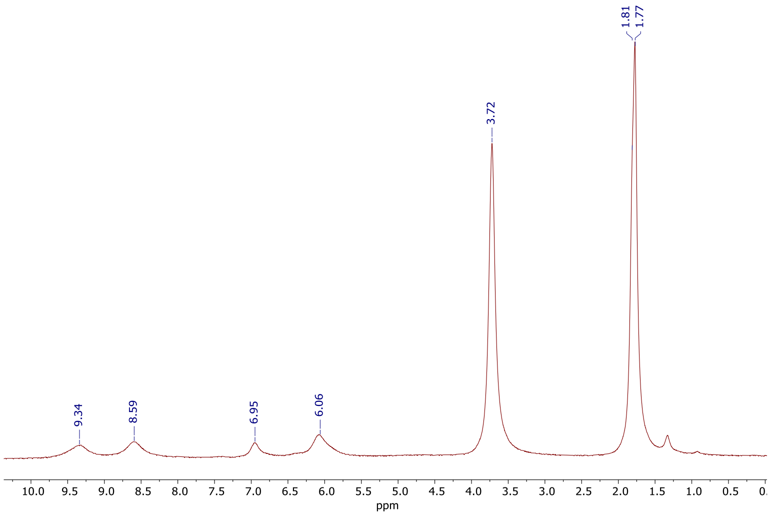

Figure S 10. ^{1}H NMR (298 K, THF-d_8, 400 MHz) spectrum of [$ErCl_3(PPO)(THF)_2$] (**4**). Note: the solvent residual peaks of THF overlaps with the signals of the coordinated THF molecules. Poor quality of NMR corresponds to the paramagnetic nature of the Er(III) centre.

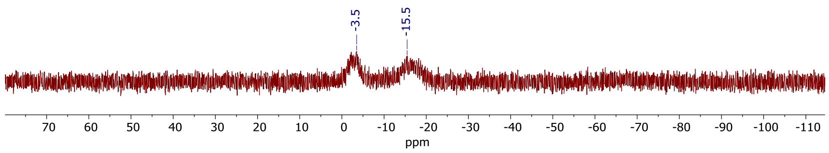

Figure S 11. $^{31}P\{^{1}H\}$ NMR (298 K, THF-d_8, 162 MHz) spectrum of [$ErCl_3$(PPO)($THF)_2$] (**4**). Note: poor quality of NMR corresponds to the paramagnetic nature of the Er(III) center.

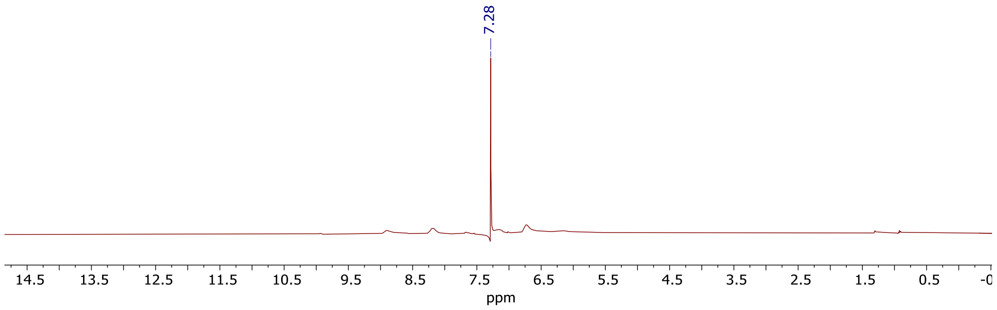

Figure S 12. ^{1}H NMR (298 K, $CDCl_3$, 400 MHz) spectrum of [$YbCl_3$(PPO)($THF)_2$] (**5**). Note: poor quality of NMR corresponds to the paramagnetic nature of the Yb(III) centre.

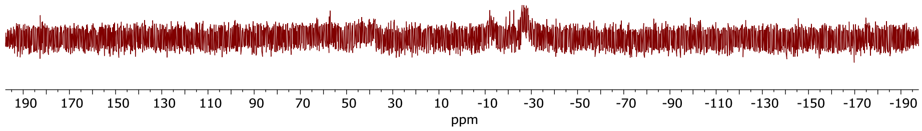

Figure S 13. $^{31}P\{^{1}H\}$ NMR (298 K, $CDCl_3$, 162 MHz) spectrum of [$YbCl_3$(PPO)($THF)_2$] (**5**). Note: poor quality of NMR corresponds to the paramagnetic nature of the Yb(III) centre.

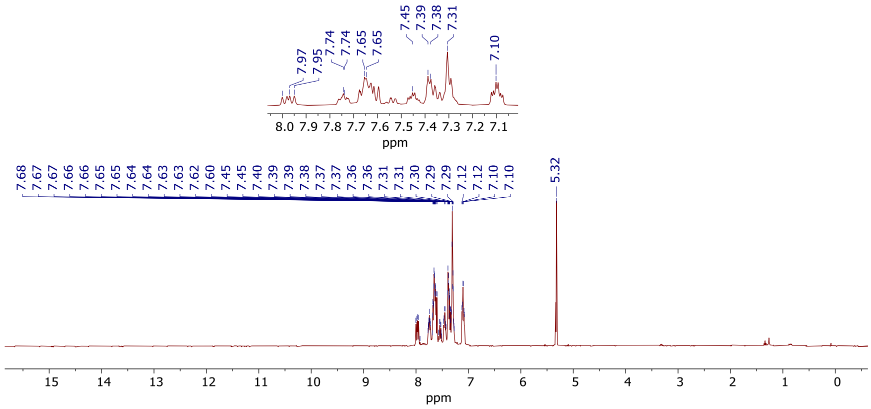

Figure S 14. ^{1}H NMR (298 K, CD_2Cl_2, 400 MHz) spectrum of [(PPO)$Cl_2Y\{\mu\text{-}Cl_3\}YCl(PPO)_2$] (**6**).

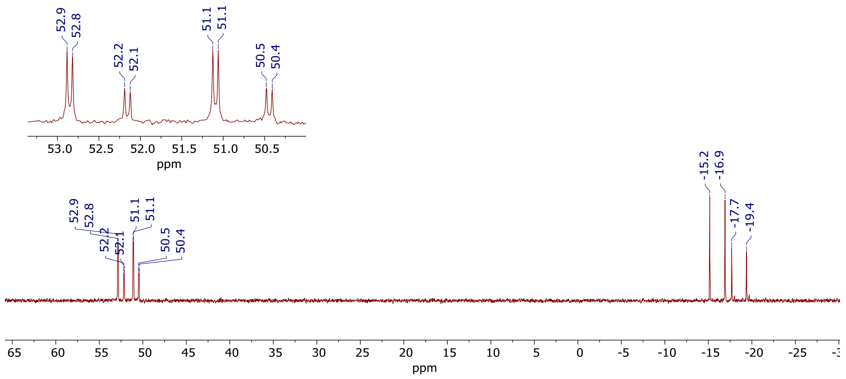

Figure S 15. $^{31}P\{^{1}H\}$ NMR (298 K, CD_2Cl_2, 162 MHz) spectrum of [(PPO)Cl_2Y{μ-Cl_3}YCl$(PPO)_2$] (**6**).

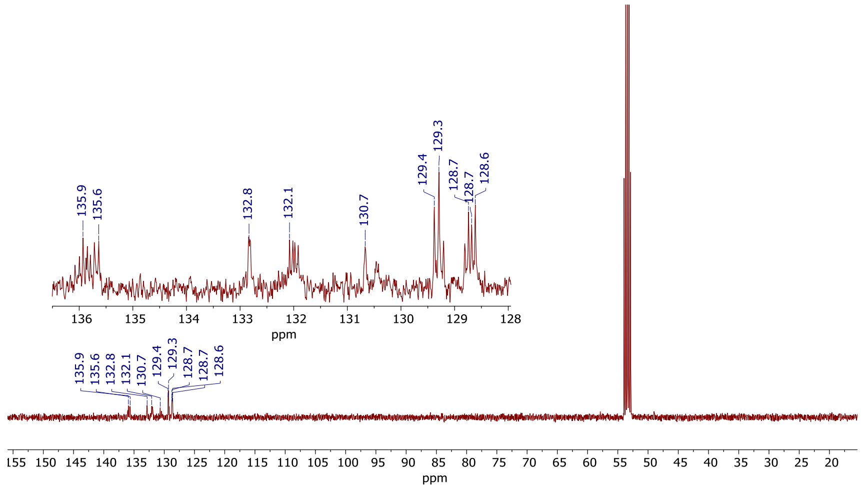

Figure S 16. $^{13}C\{^{1}H\}$ NMR (298 K, CD_2Cl_2, 101 MHz) spectrum of [(PPO)Cl_2Y{μ-Cl_3}YCl$(PPO)_2$] (**6**).

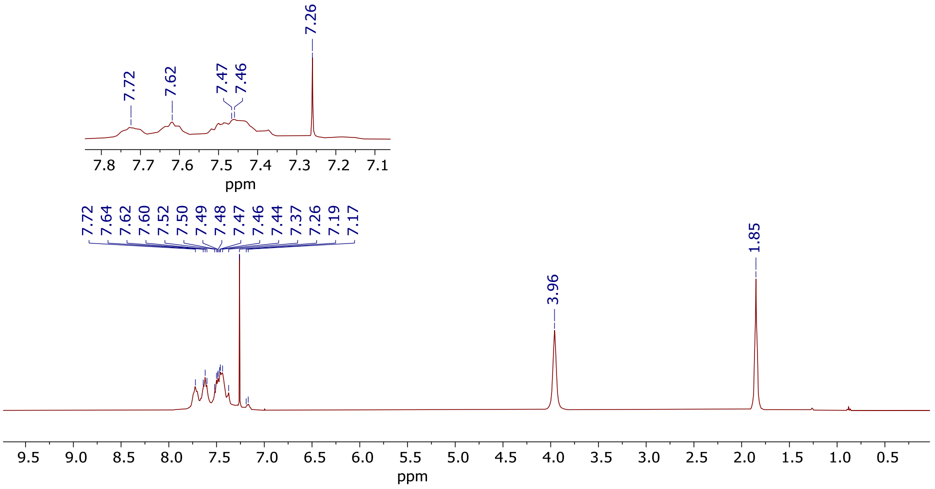

Figure S 17. ^{1}H NMR (298 K, $CDCl_3$, 400 MHz) spectrum of [YCl_3(PPO)$(THF)_2$Mo$(CO)_5$] (**7**).

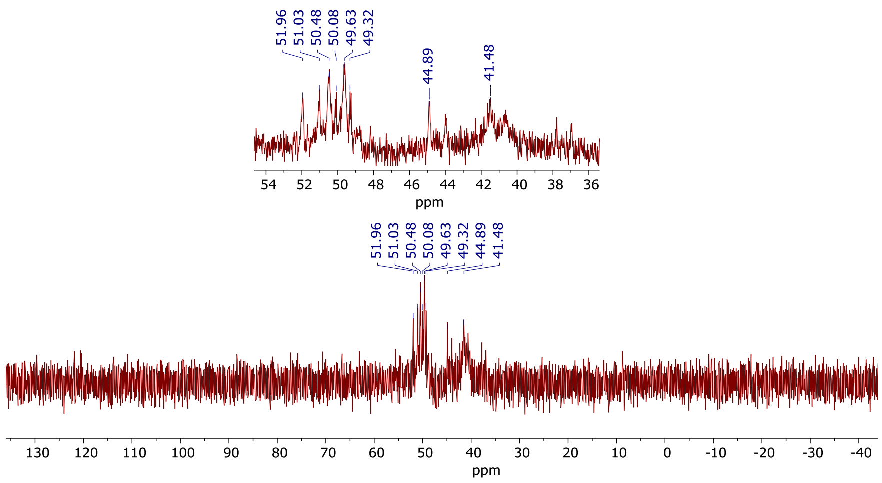

Figure S 18. $^{31}P\{^{1}H\}$ NMR (298 K, $CDCl_3$, 162 MHz) spectrum of $[YCl_3(PPO)(THF)_2Mo(CO)_5]$ (**7**).

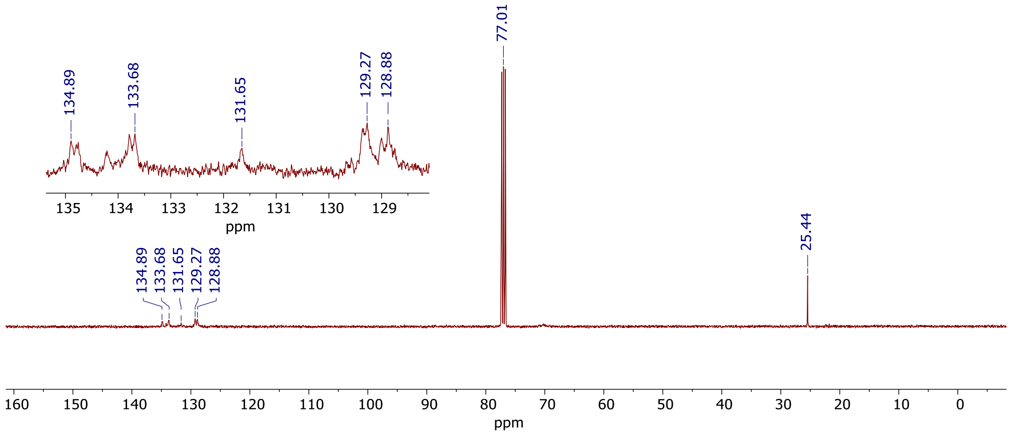

Figure S 19. $^{13}C\{^{1}H\}$ NMR (298 K, $CDCl_3$, 101 MHz) spectrum of $[YCl_3(PPO)(THF)_2Mo(CO)_5]$ (**7**). Note: Despite increasing the scan numbers a good data could not be still obtained.

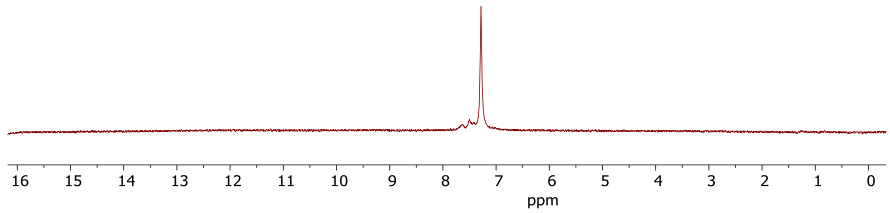

Figure S 20. ^{1}H NMR (298 K, $CDCl_3$, 400 MHz) spectrum of $[DyCl_3(PPO)(THF)_2Mo(CO)_5]$ (**8**). Note: Poor quality of NMR corresponds to the paramagnetic nature of the Dy(III) centre.

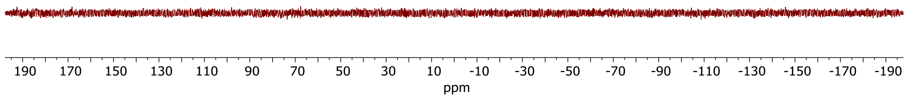

Figure S 21. $^{31}P\{^{1}H\}$ NMR (298 K, $CDCl_3$, 162 MHz) spectrum of $[DyCl_3(PPO)(THF)_2Mo(CO)_5]$ (**8**). Note: poor quality of NMR corresponds to the paramagnetic nature of the Dy(III) centre.

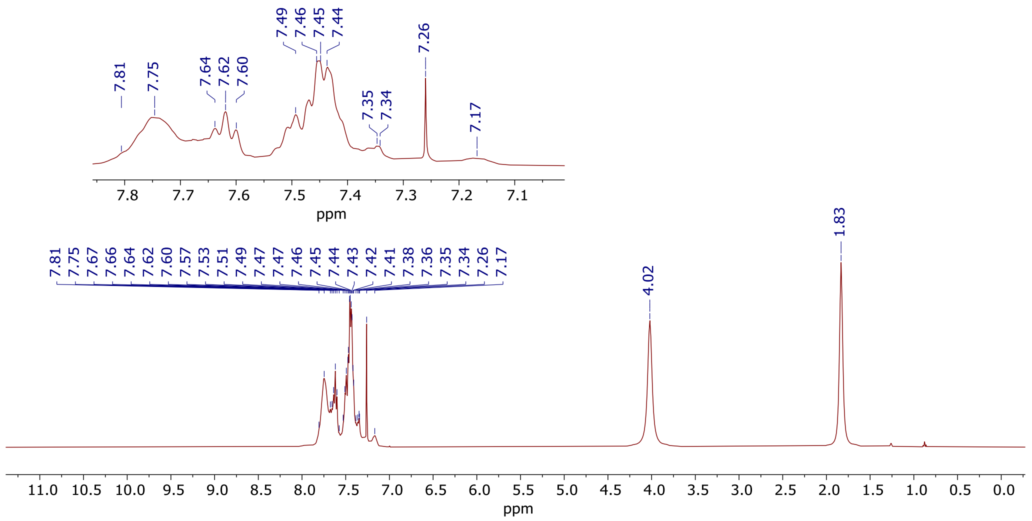

Figure S 22. ^{1}H NMR (298 K, $CDCl_3$, 400 MHz) spectrum of [$LuCl_3$(PPO)$(THF)_2$Mo$(CO)_5$] (**9**).

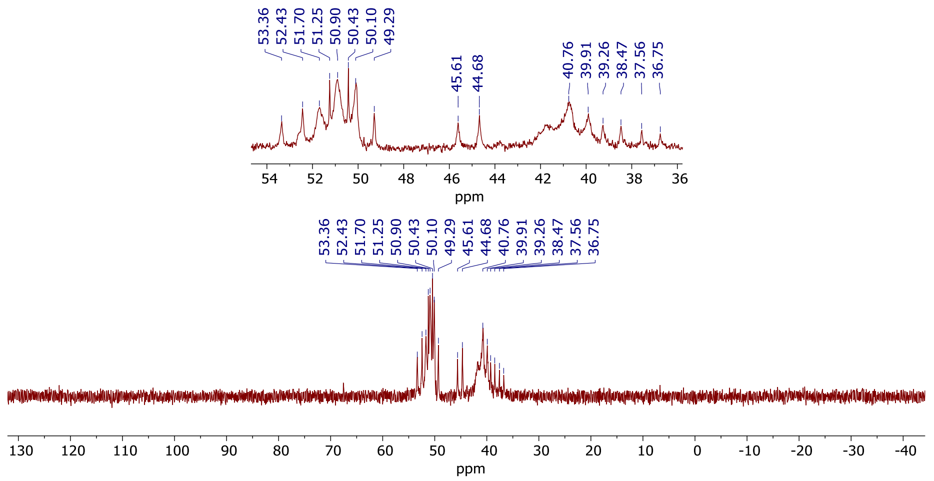

Figure S 23. $^{31}P\{^{1}H\}$ NMR (298 K, $CDCl_3$, 162 MHz) spectrum of [$LuCl_3$(PPO)$(THF)_2$Mo $(CO)_5$] (**9**).

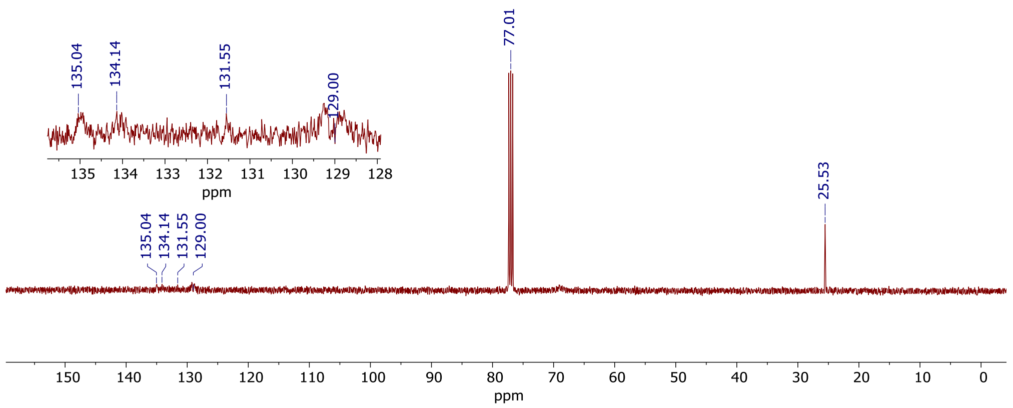

Figure S 24. $^{13}C\{^{1}H\}$ NMR (298 K, $CDCl_3$, 101 MHz) spectrum of [$LuCl_3$(PPO)$(THF)_2$Mo $(CO)_5$] (**9**). Note: Despite increasing the scan numbers a good data could not be still obtained.

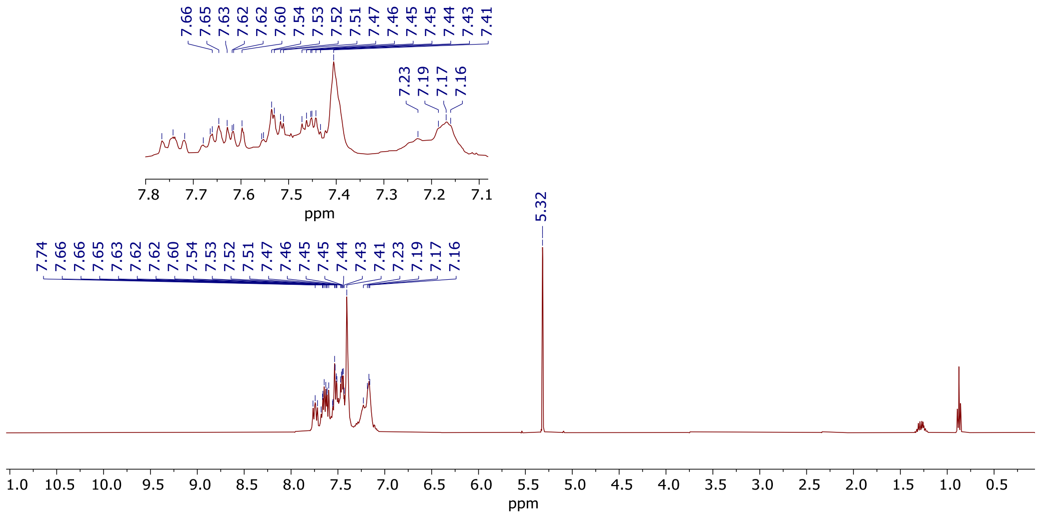

Figure S 25. ^{1}H NMR (298 K, CD_2Cl_2, 400 MHz) spectrum of [Mo(CO)$_5$(PPO)Cl_2Lu{μ-Cl_3}LuCl(PPO)$_2$(Mo(CO)$_5$)$_2$] (**10**).

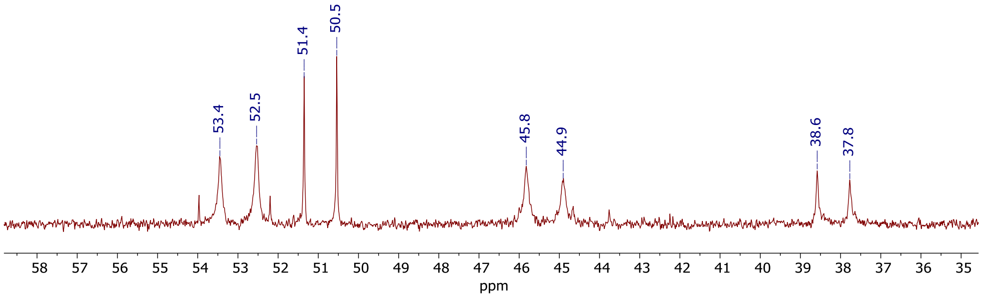

Figure S 26. ^{31}P{^{1}H} NMR (298 K, CD_2Cl_2, 162 MHz) spectrum of [Mo(CO)$_5$(PPO)Cl_2Lu{μ-Cl_3}LuCl(PPO)$_2$(Mo(CO)$_5$)$_2$] (**10**).

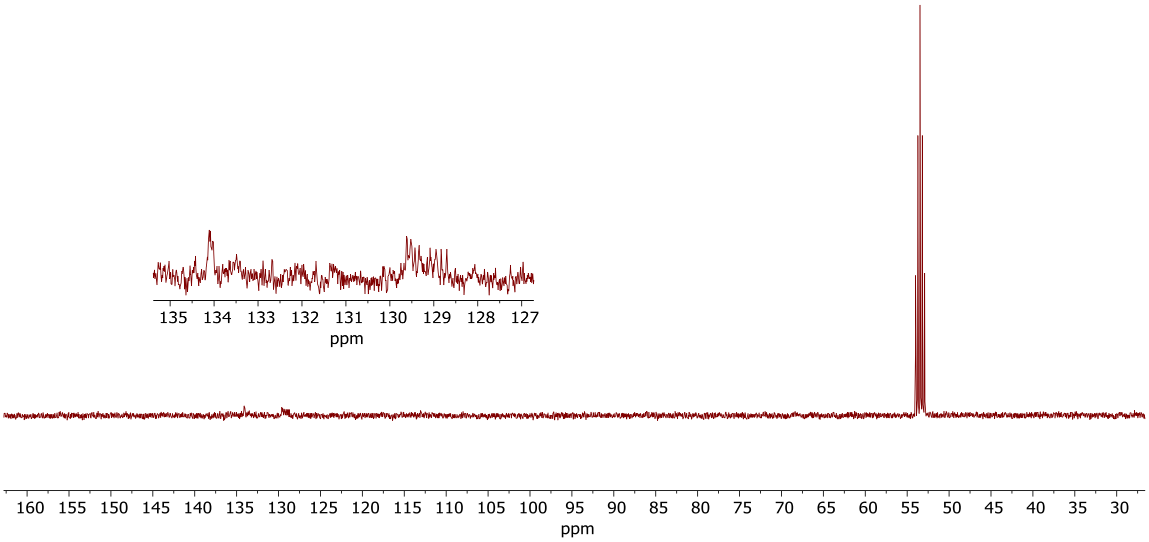

Figure S 27. ^{13}C{^{1}H} NMR (298 K, CD_2Cl_2, 101 MHz) spectrum of [Mo(CO)$_5$(PPO)Cl_2Lu{μ-Cl_3}LuCl(PPO)$_2$(Mo(CO)$_5$)$_2$] (**10**). Note: Despite increasing the scan numbers a good data could not be still obtained.

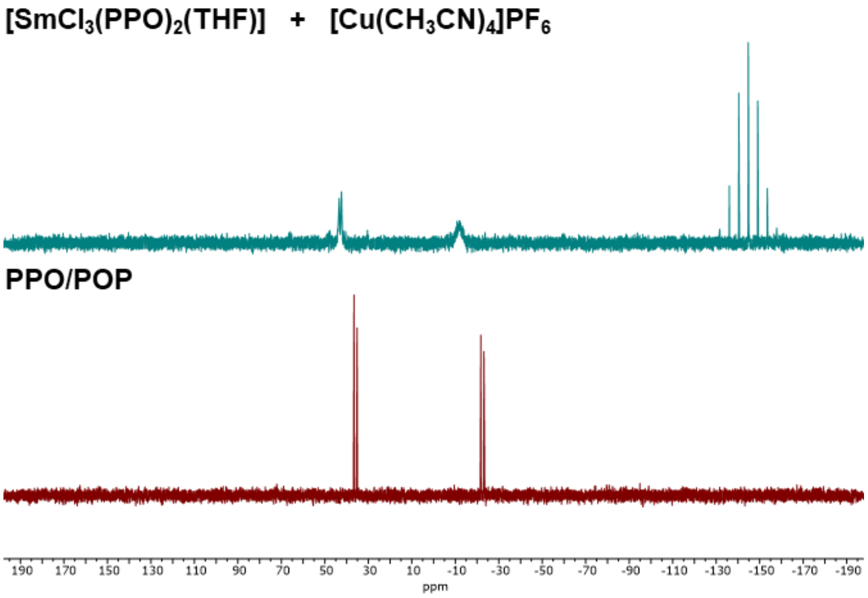

Figure S 28. $^{31}P\{^{1}H\}$ NMR spectrum (298 K, $CDCl_3$, 162 MHz) of the reaction of $[Cu(CH_3CN)_4]PF_6$ with SmPPO (**2**) (top) and $^{31}P\{^{1}H\}$ NMR spectrum (298 K, $CDCl_3$, 162 MHz) of the free PPO ligand (bottom), showing coordination of Sm to the O of P(IV) and Cu to the P(II). The slight downfield shifts and peak broadening observed in the complex relative to the free ligand indicate metal coordination at the donor sites.

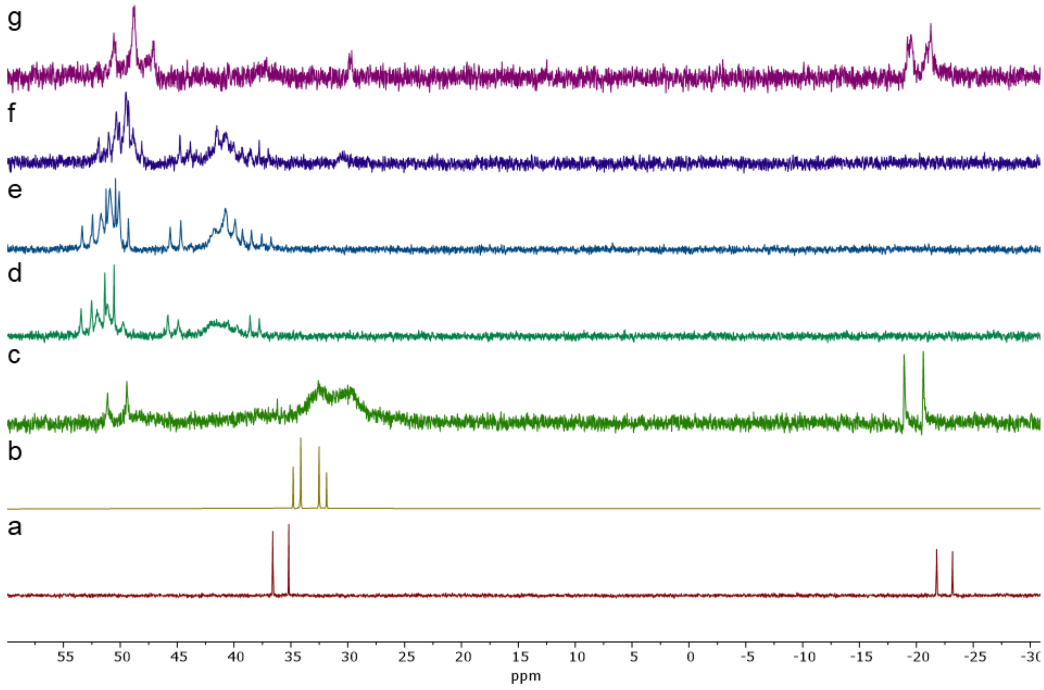

Figure S 29. $^{31}P\{^{1}H\}$ NMR spectra (298 K, 162 MHz) of a) free PPO ligand in $CDCl_3$, b) MoPPO in $CDCl_3$, complex **9** in c) THF-d_8, d) CD_2Cl_2 and e) $CDCl_3$, complex **7** in f) $CDCl_3$ and g) CD_3CN.

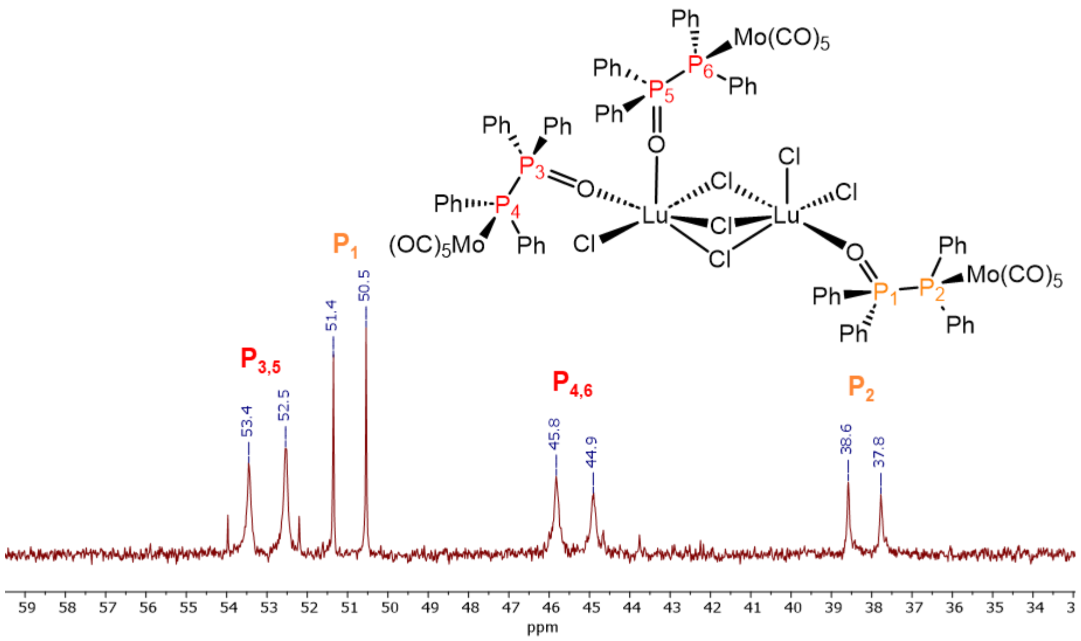

Figure S 30. $^{31}P\{^{1}H\}$ NMR (298 K, CD_2Cl_2, 162 MHz) spectrum of **10**, showing the arrangement of the dinuclear heterobimetallic assembly.

2. IR spectra

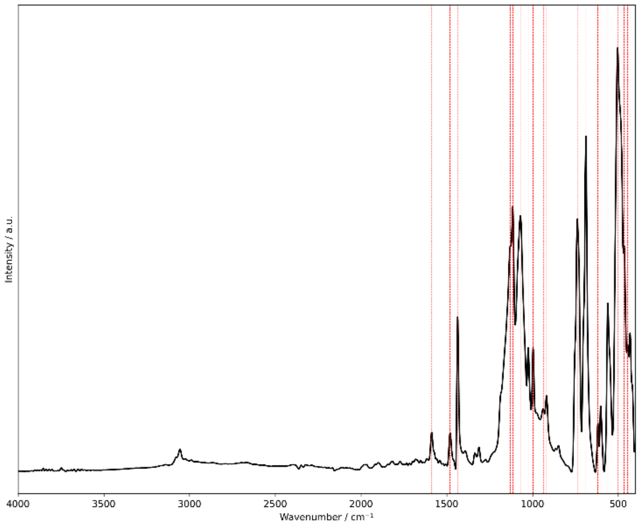

Figure S 31. IR spectrum of complex **1** recorded at 298 K in nitrogen.

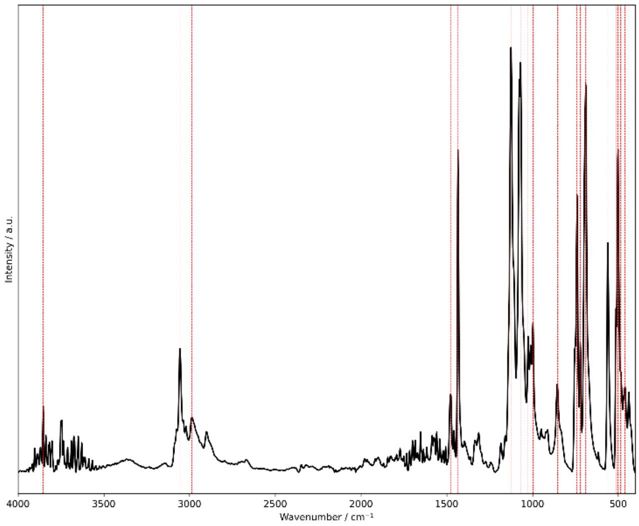

Figure S 32. IR spectrum of complex **2** recorded at 298 K in nitrogen.

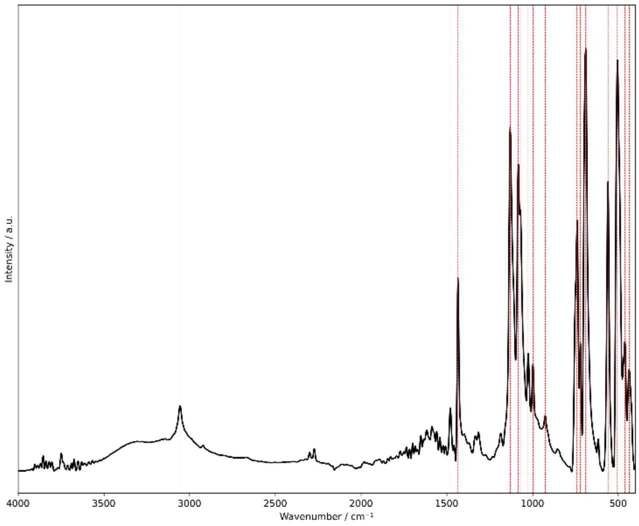

Figure S 33. IR spectrum of complex **3** recorded at 298 K in nitrogen.

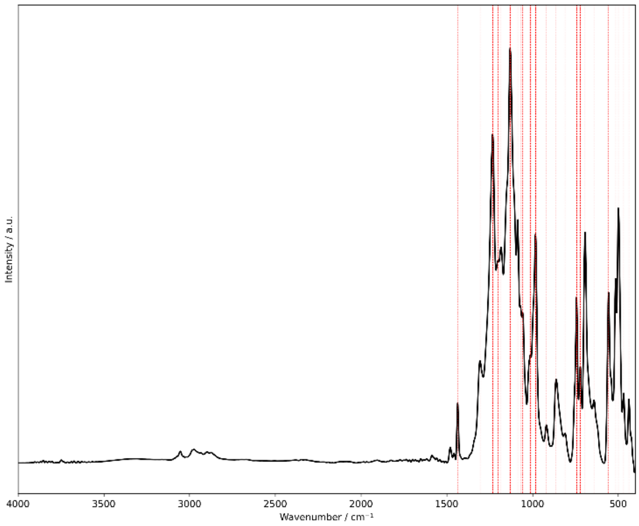

Figure S 34. IR spectrum of complex **4** recorded at 298 K in nitrogen.

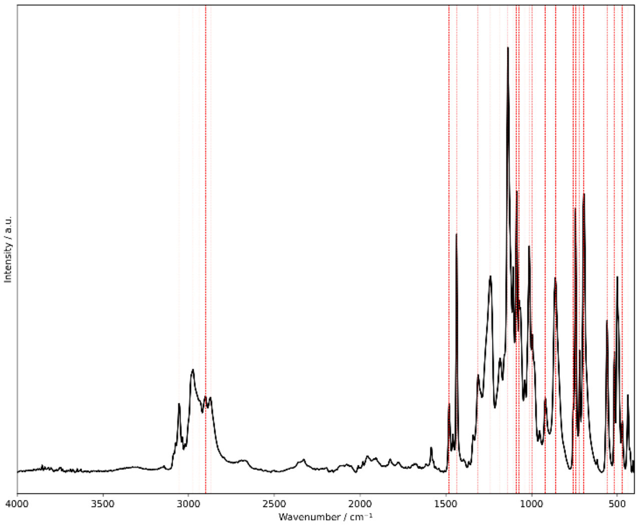

Figure S 35. IR spectrum of complex **5** recorded at 298 K in nitrogen.

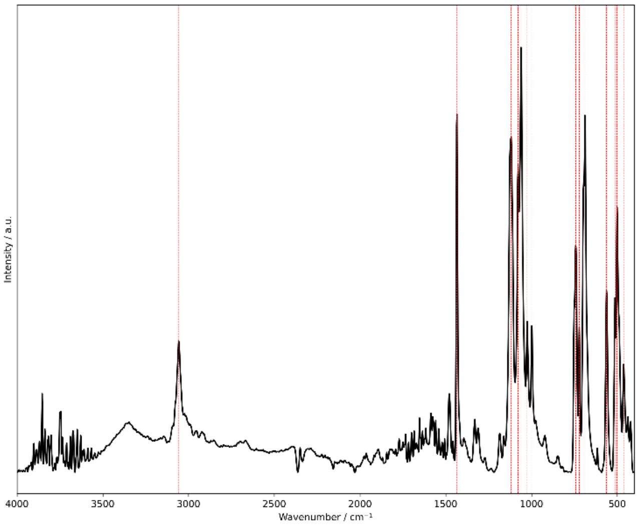

Figure S 36. IR spectrum of complex **6** recorded at 298 K in nitrogen.

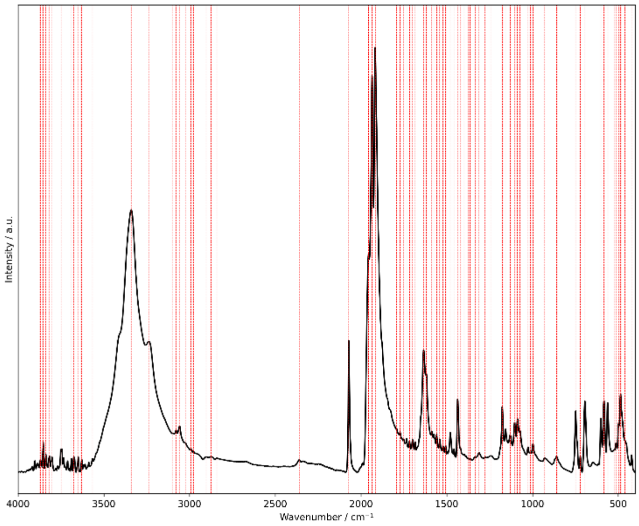

Figure S 37. IR spectrum of complex **7** recorded at 298 K in nitrogen.

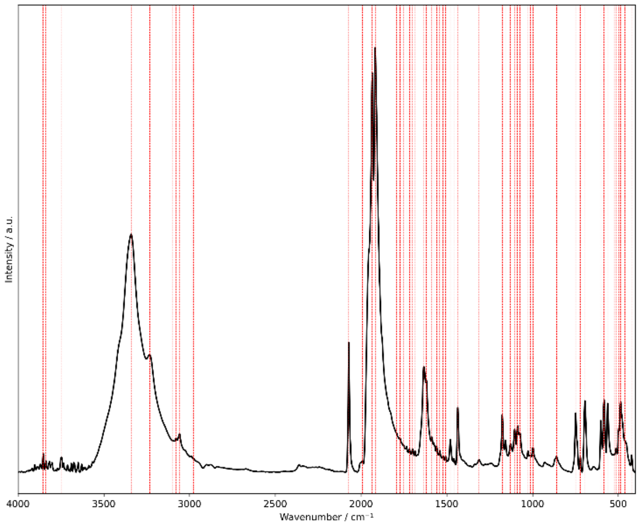

Figure S 38. IR spectrum of complex **8** recorded at 298 K in nitrogen.

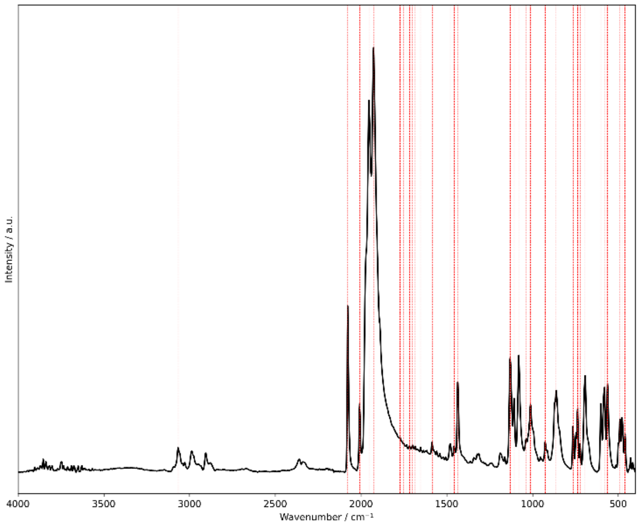

Figure S 39. IR spectrum of complex **9** recorded at 298 K in nitrogen.

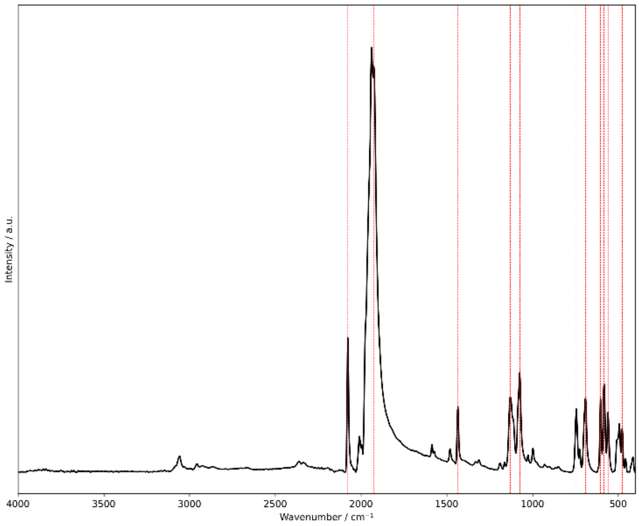

Figure S 40. IR spectrum of complex **10** recorded at 298 K in nitrogen.

3. UV-Vis spectra

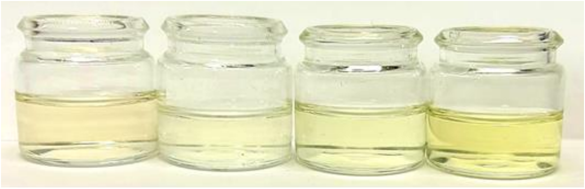

a

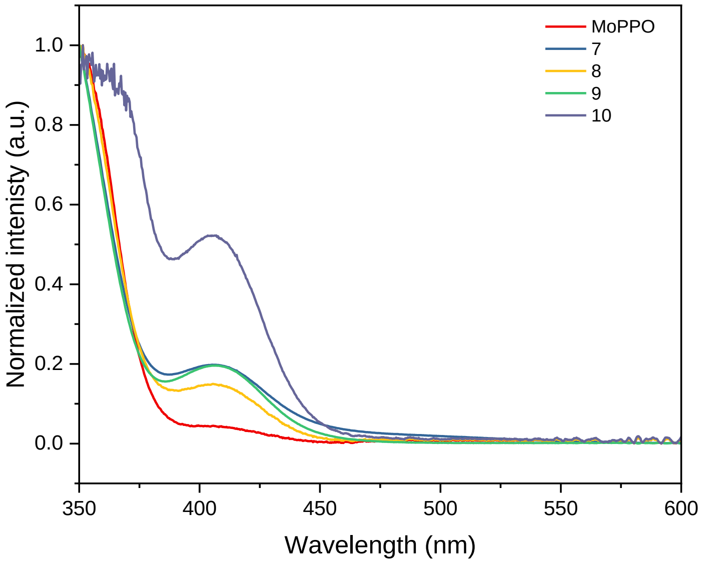

b

Figure S 41. a) Samples for UV-vis spectroscopy showing various shades of yellow colour and b) UV-vis spectra for MoPPO and complexes **7**, **8**, **9** and **10** in THF solution. (1×10^{-3} M).

Table S 1. Molar extinction coefficients determined from the UV-vis spectra, measured at a concentration of 1×10^{-3} M in THF.

**Compound**	**Wavelength λ [nm]**	**Absorbance**	**Molar extinction coefficient ε L·cm^{-1}·mol^{-1}**
MoPPO	409.1	0.078	78
**7**	407.8	0.407	407
**8**	409.1	0.301	301
**9**	406.9	0.433	433
**10**	405.2	1.506	1506

4. Single-crystal X-ray crystallography

Table S 2. Crystallographic parameters of the complexes **1-4**.

**Compound**	**1**	**2**	**3**	**4**·(0.5THF)
Formula	$C_{24}H_{20}AlCl_3OP_2$	$C_{52}H_{48}Cl_3O_3P_4Sm$	$C_{50}H_{43}Cl_3DyNO_2P_4$	$C_{32}H_{40}Cl_3ErO_{3.5}P_2$
FW / g mol^{-1}	519.67	1101.48	1082.58	840.21
Crystal System	triclinic	monoclinic	orthorhombic	triclinic
Space Group	*P*1	$P2_1/c$	$P2_12_12_1$	*P*
a / Å	7.2911(4)	10.0663(8)	9.6582(2)	10.1386(4)
b / Å	9.7964(5)	18.5729(12)	18.2016(6)	10.4318(5)
c / Å	9.9718(5)	26.708(2)	26.9187(8)	16.5731(8)
α / °	100.625(4)	90	90	87.276(4)
β / °	108.269(4)	99.052(7)	90	86.919(4)
γ / °	108.669(4)	90	90	81.015(4)
V / Å^3	607.82(6)	4931.1(7)	4732.2(2)	1727.46(14)
Z	1	4	4	2
T/K	100(2)	100(2)	100(2)	100(2)
F(000)	266	2228	2172	842
D_c/ Mg m^{-3}	1.420	1.484	1.520	1.615
μ(Mo-Kα) / mm^{-1}	0.560	1.525	1.924	2.788
Data Measured	12034	31453	30878	21363
Unique Data	5605	11224	11179	8327
R_{int}	0.0218	0.0887	0.0329	0.0355
Data with I ≥ 2σ(I)	5401	8014	9319	6792
Parameters/ Restraints	280 / 3	606 / 11	560 / 2	383 / 37
wR_2 (all data)	0.0743	0.2226	0.0614	0.0874
S (all data)	1.039	1.091	0.969	1.032
R_1 [I ≥ 2σ(I)]	0.0287	0.0839	0.0313	0.0368
Flack χ	-0.01(4)	-	-0.019(6)	-

Biggest diff. peak/ hole / eÅ^{-3}	0.34 / -0.21	1.40 / -1.75	0.69 / -0.73	1.39 / -1.74
CCDC number	2488821	2488829	2488830	2488824

Table S 3. Crystallographic parameters of the complexes **5-8**.

**Compound**	**5**·(0.5THF)	**6**	**7**	**8**
Formula	$C_{32}H_{40}Cl_3O_{3.5}P_2Yb$	$C_{72}H_{60}Cl_6O_3P_6Y_2$	$C_{37}H_{36}Cl_3MoO_8P_2Y$	$C_{37}H_{36}Cl_3DyMoO_8P_2$
FW / g mol^{-1}	845.99	1549.54	961.80	1035.39
Crystal System	triclinic	triclinic	triclinic	triclinic
Space Group	*P*	*P*	*P*	*P*
a / Å	10.1258(4)	11.9678(12)	9.9735(2)	9.9817(3)
b / Å	10.4434(3)	14.3355(16)	10.6701(3)	10.6617(3)
c / Å	16.6238(6)	21.837(2)	21.3365(5)	21.3668(6)
α / °	86.845(3)	72.915(8)	86.073(2)	86.150(3)
β / °	86.773(3)	81.769(8)	81.520(2)	81.480(2)
γ / °	80.933(3)	80.847(8)	64.819(2)	64.844(2)
V / Å^3	1731.31(11)	3517.0(7)	2032.31(9)	2035.49(11)
Z	2	2	2	2
T/K	100(2)	100(2)	100(2)	100(2)
F(000)	846	1572	968	1022
D_c/ Mg m^{-3}	1.623	1.463	1.572	1.689
μ(Mo-Kα) / mm^{-1}	3.059	2.052	2.053	2.453
Data Measured	25440	35984	28840	28885
Unique Data	8844	13770	10228	10497
R_{int}	0.0996	0.0541	0.0364	0.0665
Data with I ≥ 2σ(I)	7249	9509	8616	8366
Parameters/ Restraints	371 / 0	820 / 288	470 / 0	469 / 0
wR_2 (all data)	0.1079	0.1524	0.0647	0.0654
S (all data)	0.985	1.048	1.019	0.959

R_1 [I ≥ 2σ(I)]	0.0446	0.0593	0.0280	0.0318
Flack χ	-	-	-	
Biggest diff. peak/ hole / eÅ^{-3}	1.56 / -2.53	1.52 / -0.87	0.37 / -0.61	0.90 / -1.34
CCDC number	2488823	2488825	2488822	2488828

Table S 4. Crystallographic parameters of the complexes **9** and **10**.

**Compound**	**9**	**10**·(3DCM)·(1*n*-pentane)
Formula	$C_{37}H_{36}Cl_3LuMoO_8P_2$	$C_{95}H_{78}Cl_{12}Lu_2Mo_3O_{18}P_6$
FW / g mol^{-1}	1047.86	2756.55
Crystal System	triclinic	triclinic
Space Group	*P*	*P*
a / Å	9.9478(5)	13.9840(4)
b / Å	10.6440(6)	18.2529(5)
c / Å	21.2839(10)	21.4746(6)
α / °	86.012(4)	91.402(2)
β / °	81.470(4)	101.812(2)
γ / °	64.928(4)	95.965(2)
V / Å^3	2018.65(19)	5330.3(3)
Z	2	2
T/K	100(2)	100(2)
F(000)	1032	2708
D_c/ Mg m^{-3}	1.724	1.717
μ(Mo-Kα) / mm^{-1}	3.068	2.631
Data Measured	27195	59665
Unique Data	9946	26278
R_{int}	0.0577	0.0312
Data with I ≥ 2σ(I)	8083	19524
Parameters/Restraints	470 / 0	1133 / 3
wR_2 (all data)	0.0586	0.1201
S (all data)	0.947	1.048
R_1 [I ≥ 2σ(I)]	0.0300	0.0442
Flack χ		

Biggest diff. peak/ hole / eÅ^{-3}	1.13 / -1.38	2.04 / -2.14
CCDC number	2488827	2488826

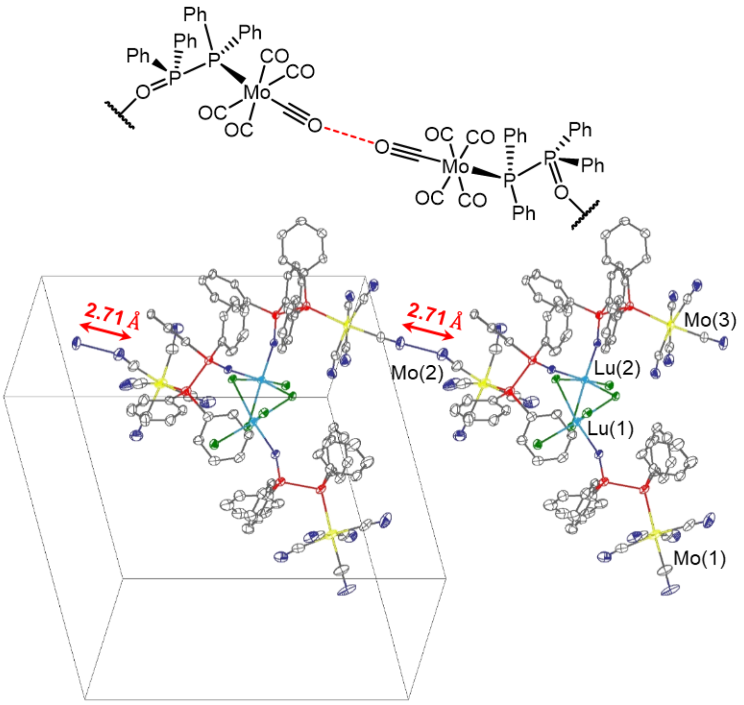

Figure S 42. Molecular structure of [Mo(CO)$_5$(PPO)Cl$_2$Lu{μ-Cl$_3$}LuCl(PPO)$_2$Mo(CO)$_5$] (**10**), showing the interaction between two adjacent carbonyl oxygen atoms of Mo–CO.
